# Evaluation of the Antimicrobial Activity in Host-Mimicking Media and *In Vivo* Toxicity of Antimicrobial Polymers as Functional Mimics of AMPs

**DOI:** 10.1021/acsami.2c05979

**Published:** 2022-07-12

**Authors:** Ramón Garcia Maset, Alexia Hapeshi, Stephen Hall, Robert M. Dalgliesh, Freya Harrison, Sébastien Perrier

**Affiliations:** † Warwick Medical School, University of Warwick, Coventry CV4 7AL, U.K.; ‡ Department of Chemistry, University of Warwick, Coventry CV4 7AL, U.K.; § ISIS Neutron and Muon Source, Rutherford Appleton Laboratory, Didcot OX11 0DE, U.K.; ∥ School of Life Sciences, University of Warwick, Coventry CV4 7AL, U.K.; ⊥ Faculty of Pharmacy and Pharmaceutical Sciences, Monash University, Parkville, Victoria 3052, Australia

**Keywords:** antimicrobial, AMP mimics, cationic polymers, *Galleria mellonella*, wound infection, cystic fibrosis

## Abstract

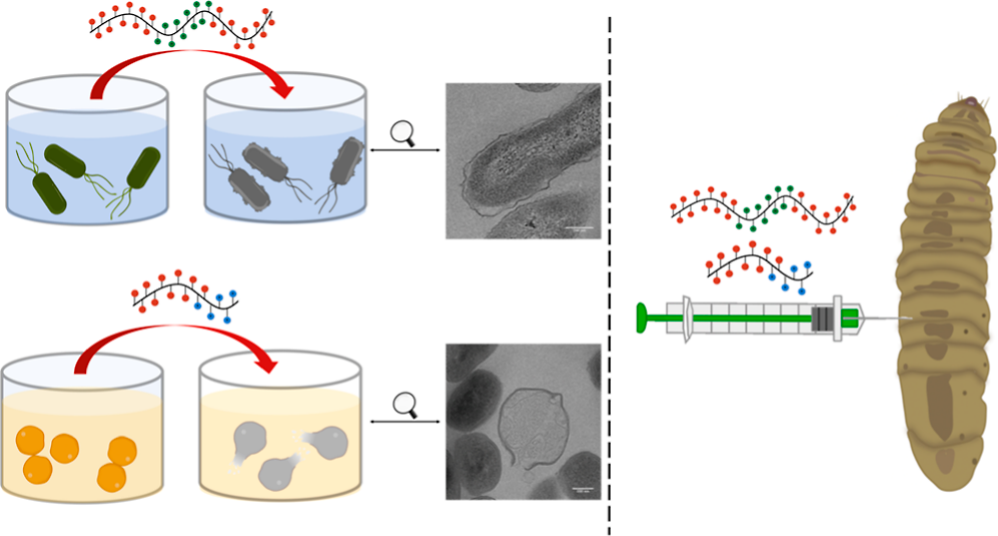

Activity tests for synthetic antimicrobial compounds are often limited to the minimal inhibitory concentration assay using standard media and bacterial strains. In this study, a family of acrylamide copolymers that act as synthetic mimics of antimicrobial peptides were synthesized and shown to have a disruptive effect on bacterial membranes and structural integrity through microscopy techniques and membrane polarization experiments. The polymers were tested for their antimicrobial properties using media that mimic clinically relevant conditions. Additionally, their activity was compared in two different strains of the Gram-positive bacterium *Staphylococcus aureus* and the Gram-negative bacterium *Pseudomonas aeruginosa*. We showed that the medium composition can have an important influence on the polymer activity as there was a considerable reduction in minimal inhibitory concentrations against *S. aureus* grown in synthetic wound fluid (SWF), and against *P. aeruginosa* grown in synthetic cystic fibrosis sputum media (SCFM), compared to the concentrations in standard testing media. In contrast, we observed a complete loss of activity against *P. aeruginosa* in the serum-containing SWF. Finally, we made use of an emerging invertebrate *in vivo* model, using *Galleria mellonella* larvae, to assess toxicity of the polymeric antimicrobials, showing a good correlation with cell line toxicity measurements and demonstrating its potential in the evaluation of novel antimicrobial materials.

## Introduction

The World Health Organization (WHO) has identified the antimicrobial resistance (AMR) crisis as one of the most pressing issues of our times.^1^ Multidrug-resistant bacteria are one of the major causes of death globally (5 million deaths in 2019), jeopardizing the effectiveness of modern medicine.^2^ Infections caused by the *ESKAPE* pathogens (*i.e.,Enterococcus faecium*, *Staphylococcus aureus*, *Klebsiella pneumoniae*, *Acinetobacter baumannii*, *Pseudomonas aeruginosa*, and *Enterobacter* species) are especially concerning for their high mortality risk.^3^ In the last 20 years, only six new classes of antibiotics have reached the clinic, and none of them were active against Gram-negative bacteria.^4^

Bacterial pathogens are able to quickly evolve resistance against conventional small molecule antibiotics with single cellular targets or modes of action. This often arises from the modification of the target molecules in the cell. Inspired by nature, researchers have focused on antimicrobial peptides (AMPs) as an alternative attempt to tackle the global antimicrobial crisis.^5^ AMPs are short amphipathic amino acid chains with cationic and hydrophobic moieties,^6^ which possess broad antimicrobial activity against Gram-positive and Gram-negative bacteria, fungi, viruses, and parasites. One of the predominant mechanisms of action of AMPs is the disruption of the bacterial membrane integrity. This is mediated by electrostatic interactions between the negatively charged moieties on the bacterial surface with the positively charged residues within AMPs, combined with the hydrophobic AMP side chains driving insertion into the bacterial membrane.^7^ Additionally, AMPs have been observed to translocate through the membrane to the cytoplasm, where the binding to intracellular targets such as DNA, RNA, and proteins, leads to bacterial cell death.^8^ The presence of multiple targets, together with the large fitness cost incurred by changes in components of the cell surface, makes the emergence of resistance against AMPs rare.^9^

Even though AMPs are promising candidates as antimicrobial agents, cytotoxic effects against mammalian cells have been reported,^10^ due to their inherent ability to disrupt lipid bilayers and, therefore, cell membranes. Their application is further hindered by their proteolytic instability^11^ and the high cost associated with their synthesis.^12^ The limitations of AMPs have resulted in an increasing interest in the development of synthetic antimicrobial peptides (sAMPs). Common strategies of sAMP development include single amino acid substitutions, segmentation of AMPs to obtain smaller active fragments, chimera generation, the incorporation of unnatural amino acids, and in silico methods to predict new synthetic peptides.^13,14^ More recently, polymeric materials have also been exploited as AMP mimics in order to overcome some of the abovementioned limitations. The recent development of precision polymer synthetic methodologies and, in particular, radical-based techniques, such as reversible addition–fragmentation chain transfer (RAFT) polymerization,^15^ has allowed the precise design and synthesis of complex macromolecules, controlling the size, segmentation of block copolymers,^16^ the architecture (*e.g.*, stars, brushes, or nanoparticles),^17−19^ and their functionalization with highly diverse chemical moieties, which mimic the amino acid residues present in AMPs. Since the hydrophobicity and cationic content of AMPs play a crucial role in their antimicrobial activity, the influence of these parameters on the activity and toxicity of polymeric AMP mimics has been studied by varying the monomer types, the ratio between hydrophobic and cationic units, the segmentation of hydrophobic and cationic moieties, and the polymer architecture.^20−23^

Studies of antimicrobial activity are usually conducted by determination of the minimal inhibitory concentration (MIC) against bacteria grown planktonically in standard media. The main benefit of this assay is the direct comparison of antimicrobial activity of antibiotics across clinical and research laboratories. However, it has been observed that compounds that appear to be active under these conditions are not necessarily efficacious in a more physiological environment; conversely, some compounds with very high MIC values in standard media are actually active in host-mimicking media or *in vivo*.^24^ For example, salt concentration can influence the antimicrobial activity of AMPs as it can affect the initial electrostatic interaction with the bacterial surface. In particular, it was suggested that an increased salt concentration in the lung environment of cystic fibrosis (CF) patients^25^ could reduce the effectiveness of cathelicidin against *P. aeruginosa* infections.^26^ Moreover, it has been reported that physiological levels of divalent cations, such as Ca^2+^ and Mg^2+^, reduce the antimicrobial activity of several mammalian AMPs, especially against Gram-negative bacteria.^27^ This is at least in part due to the high affinity and stabilizing effect of divalent cations for bacterial lipopolysaccharides (LPSs) found on the surface layers of the outer membrane.^28^ Therfore, assessing the antimicrobial activity under conditions that mimic the bacterial infection environment can be one way to prevent the discrepancies between *in vitro* and *in vivo* data and reduce the chances of failure in clinical trials.^24,29^

In the present study, we investigated a family of cationic acrylamide copolymers by modifying the length and the segmentation of the hydrophobic and cationic blocks and the type of cationic moiety. We investigated their antimicrobial activity in standard cation-adjusted Müller-Hinton broth (caMHB) to allow direct comparison with the existing antimicrobials, as well as in synthetic wound fluid (SWF) and synthetic cystic fibrosis sputum medium (SCFM) in order to better mimic the environments of bacterial infections in chronic wounds and CF lungs, respectively. We reported the effectiveness of the polymers under these conditions and their cytotoxic effect *in vitro* and using an invertebrate *in vivo* model. Using two lead compounds, we demonstrated that the copolymers act by compromising the surface integrity of both Gram-negative and Gram-positive bacteria, functioning as effective AMP mimics.

## Results and Discussion

### Synthesis and Characterization of Ammonium and Guanidinium Polymers *via* RAFT Polymerization

For this study, a total of six copolymers mimicking AMPs were synthesized *via* RAFT polymerization by modifying the type of cationic group and the monomer distribution along the polymeric chain as previously reported by Kuroki *et al*.^23^ We used acrylamide monomers, namely, (guanidino-ethyl)acrylamide (GEAM) and *N*-(2-aminoethyl)acrylamide (AEAM), as the cationic moieties mimicking arginine and lysine, respectively. *N*-Isopropylacrylamide (NIPAM) was also used to introduce hydrophobicity to the system.

**Figure 1:**
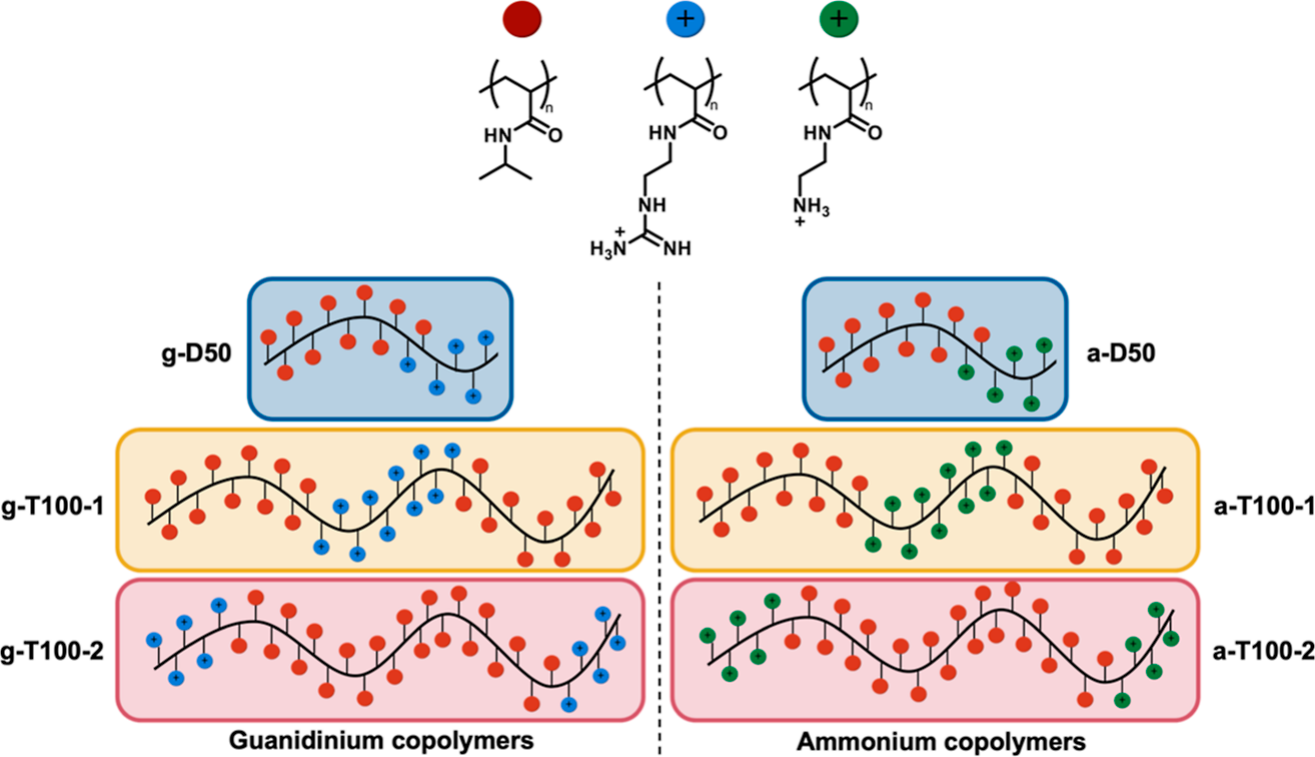
Schematic representation of the cationic copolymers (red dot for the NIPAM block, blue dot for the GEAM block, and green dot for the AEAM block).

A constant ratio of [NIPAM]/[cationic monomer] = 70:30 was maintained, independently of the molecular weight of the final polymers, since a 30% cationic ratio has been reported as ideal to provide good antimicrobial activity and high biocompatibility toward mammalian cells.^21^ For ease of reference, the polymers are identified by the type of cationic charge (“g” for guanidinium and “a” for ammonium), the degree of polymerization (DP = 50 or 100), the block sequence distribution (“D” for diblock, “T” for triblock, with the composition of the middle block identified as “T-1” for a middle positively charged segment, and “T-2” for a middle hydrophobic segment), and labeled “Boc” when in their protected form (Figure 1).

As can be observed from the ^1^H NMR spectra for the guanidinium and ammonium copolymers (Figures S3–S5), full conversion was reached for the polymerization of the first monomer, thus allowing chain extension with the addition of the second monomer in a one-pot process, without intermediate purification steps. For all the synthesized polymers, full conversion of each block was reached before further chain extensions (Figures S6–S8). All polymers showed low dispersity values (D̵ ≤ 1.21) according to SEC analyses (Figure S9 and Table S2). The shift of the molecular weight to higher values and the minimal broadening in the SEC traces demonstrated the success of the chain extension reactions in a control manner.

**Figure 2:**
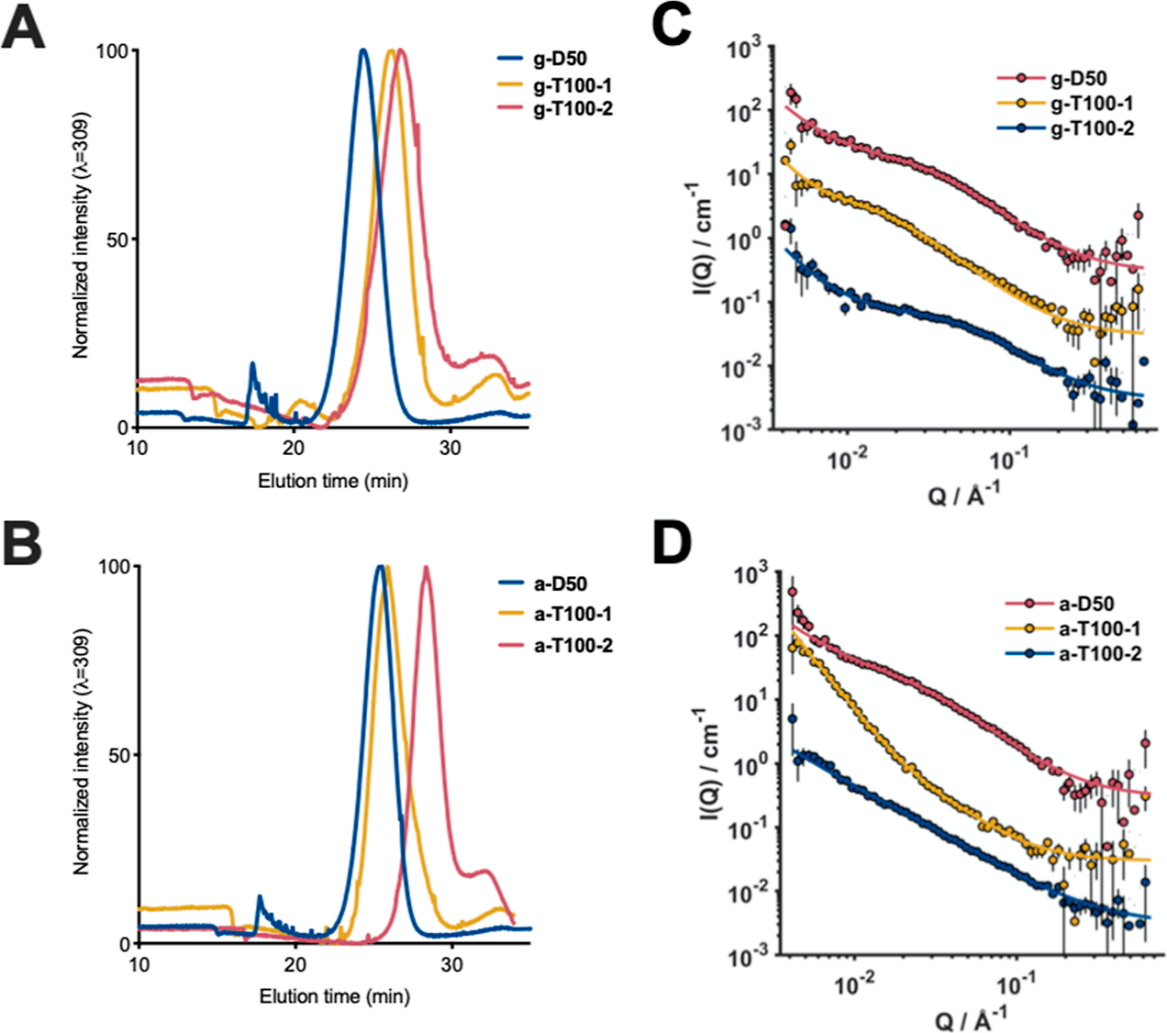
HPLC chromatograms of the guanidinium polymers (A) and the ammonium polymers (B) with a gradient of 5–95% ACN in 30 min using the 100 mm C18 column. (C) SANS data guanidinium polymers and (D) SANS data ammonium polymers.

Since it is known that the monomer distribution and the segmentation in the polymeric chain have an effect on the physicochemical properties of the polymers, we investigated the overall hydrophobicity of the synthesized compounds. For instance, hydrophobicity is a key parameter that increases the antimicrobial activity of polymeric materials, but it is also correlated with toxicity toward mammalian cells.^20,21^ The hydrophobicity of the polymers was evaluated *via* reverse-phase HPLC (RP-HPLC), following a protocol previously reported by our group.^23^ As reported by Kuroki *et al.*, guanidinium-NIPAM copolymers showed a less hydrophobic profile with increasing block segmentation. Similar to previous studies, we have observed that the more hydrophilic diblock copolymers (D50) were eluted first due to a shorter hydrophobic block in comparison to the triblock copolymers. In the case of the triblock copolymers, the difference of hydrophobicity could be related to the segregation of the hydrophobic content. For instance, T-100-2 copolymers were less segregated than T-100-1 copolymers, partially explaining the later elution time indicating greater hydrophobicity. A similar hydrophobicity pattern was observed for the guanidinium and ammonium copolymers (Figure 2A,B). The retention time of the copolymers can be found in Table S2.

Given the difference in hydrophobicity of the different polymer architectures, as evidenced by differences in the RP-HPLC retention time, it is feasible that the intrinsic amphipathic nature of these polymers could lead to self-assembly under physiologically relevant conditions. The polymers were dissolved to a concentration of 1 mg mL^–1^ in phosphate-buffered saline (PBS, pH = 7.4) and analyzed by DLS at 37 °C. In all cases, particle size distributions revealed hydrodynamic diameters below 10 nm (Figure S16), suggesting minimal self-assembly. Due to the assumption made throughout DLS analysis that all particles in solution form spherical structures, these data do not exclude the possibility of other types of aggregation in solution. Therefore, to obtain more precise structural details of the conformation of these polymers in solution, small-angle neutron scattering (SANS) measurements were performed (Figure 2C,D). In the case of guanidinium-containing copolymers, clear Gaussian-coil form factors were identified in all SANS profiles. g-D50 had the smallest radius of gyration of all polymers measured (Table S3), consistent with the low molecular weight and hydrophilicity. An increase in the scattering intensity at low momentum transfer (*Q*), however, indicated a degree of aggregation in solution occurring for each polymer and, in particular, a mass-fractal-like aggregation forming cross-linked networks of Gaussian coils. Ammonium-containing polymers, on the other hand, showed a much stronger aggregation, particularly for a-D50 and a-T100-1. It should be noted, however, that the concentrations required for the SANS experiments were much higher (5 mg mL^–1^) than those tested for biological activity, so aggregation may not greatly influence activity.

While the behavior in solution to this point has been characterized under physiologically relevant conditions, polyNIPAM has well-characterized thermoresponsive properties, possessing a lower critical solution temperature (LCST) around 32 °C in water, which is close to physiological temperatures.^30^ Previous studies have identified that copolymerization of NIPAM with a hydrophilic monomer can increase the LCST.^31^ An LCST behavior around 37 °C (physiological temperature) could affect the antimicrobial activity of the copolymers. Therefore, we investigated the solution behavior of all polymers *via* turbidity measurements. Polymer solutions (1 mg mL^–1^) in PBS (pH = 7.4) were prepared and then subjected to two heating/cooling cycles from 25 to 60 °C, and turbidity was monitored at a wavelength of 633 nm. As can be observed in Figure S17, all polymers showed a transmittance close to 100% over the whole examined temperature range, indicating no decrease in solubility with increasing temperature. This showed that the thermoresponsiveness of the pNIPAM block was completely hindered by the presence of the positively charged blocks. Therefore, the performance of the copolymers in the biological assays would not be influenced by temperature.

### Antibacterial Activity of the Cationic Acrylamide Polymers under Standard and Clinically Relevant Conditions

The antimicrobial activity of the copolymers was screened against the Gram-positive *S. aureus* and the Gram-negative *P. aeruginosa*. Both species are members of the high-priority ESKAPE group,^3^ and both are flexible opportunists, associated with a wide range of acute and chronic infections. For instance, *S. aureus* and *P. aeruginosa* are the main pathogens present in chronic wound infections.^32^ In lung infections, especially in CF patients, *P. aeruginosa* is the most prevalent pathogen.^33^*Pseudomonas* bacteria were shown to have a different pattern of gene expression and phenotypic characteristics in lung sputum or in media mimicking the composition of CF sputum compared to when grown in standard laboratory media.^34^ This includes changes in membrane composition, which can affect antibiotic susceptibility.^35^ Since the media can have an effect on the bacterial physiology and on the antimicrobial activity, we investigated the antimicrobial activity under standard conditions and with growth media that mimic the environment of two types of infections: (i) a chronic wound infection using SWF^36^ and (ii) a respiratory infection of a CF lung using SCFM.^34^

We used two isolates of each species. On the one hand, *S. aureus* Newman (ATCC 25904) is a well-studied laboratory strain, but it is known to be a relatively weak biofilm former and relatively sensitive to antibiotic treatment.^37^ On the other hand, *S. aureus* USA300 LAC is a community-associated MRSA clone also known to be a good biofilm former.^38^*P. aeruginosa* PA14 is widely used as a reference strain, and it is known to have high acute virulence and strong biofilm formation.^39^*P. aeruginosa* LESB58 is an isolate from the Liverpool epidemic strain, a transmissible clone which causes significant disease in people with CF, and it is often used as a representative CF isolate.^40^

First, we investigated the antimicrobial activity of the polymers using the broth microdilution method with standard testing medium (cation-adjusted Müller-Hinton broth, caMHB). In the case of both *S. aureus* strains, the MIC values evidenced a clear correlation between the antimicrobial activity and the type of cationic charge. Guanidinium copolymers showed a higher antimicrobial activity (lower MIC values) than their ammonium counterparts against *S. aureus* strains (Table 1). Moreover, triblock copolymers showed an improved antimicrobial activity than lower molecular weight diblock copolymers. In the case of *P. aeruginosa* strains, no influence of the cationic moieties on the antimicrobial activity was observed in PA14, while ammonium copolymers were slightly more active than their guanidinium counterparts against LESB58. Strikingly, a-D50 and g-D50 were completely inactive even at high concentration (∼38 μM and 512 μg mL^–1^) against *P. aeruginosa* PA14 (Table 1). In contrast, in the case of the clinical isolate LESB58, both polymers were active, evidencing a difference between clinical isolates and laboratory strains (Table 1). The LESB58 strain possesses a rough LPS devoid of the O-antigen in the outer membrane in comparison with the smooth LPS (with the O-antigen) of PA14 strain.^41^ We hypothesized that the difference observed between the diblock copolymer against the two strains might be related with the type of LPS, since the copolymer was likely to interact with LPS by electrostatic interaction as other AMPs, such as colistin or polymyxin B.^42^ The activity of the compounds in the standard medium was influenced to some extent by the nature of the charge. However, a significant difference between the activity of the diblock copolymers and that of the longer triblock copolymers could be observed. Previously, we demonstrated that multiblock copolymers were more efficient antimicrobials compared to their statistical or diblock equivalents,^21^ while other authors showed that an increase in the molecular weight could result in improved activities of statistical guanidinium-based methacrylate copolymers.^43^

**Table 1:**
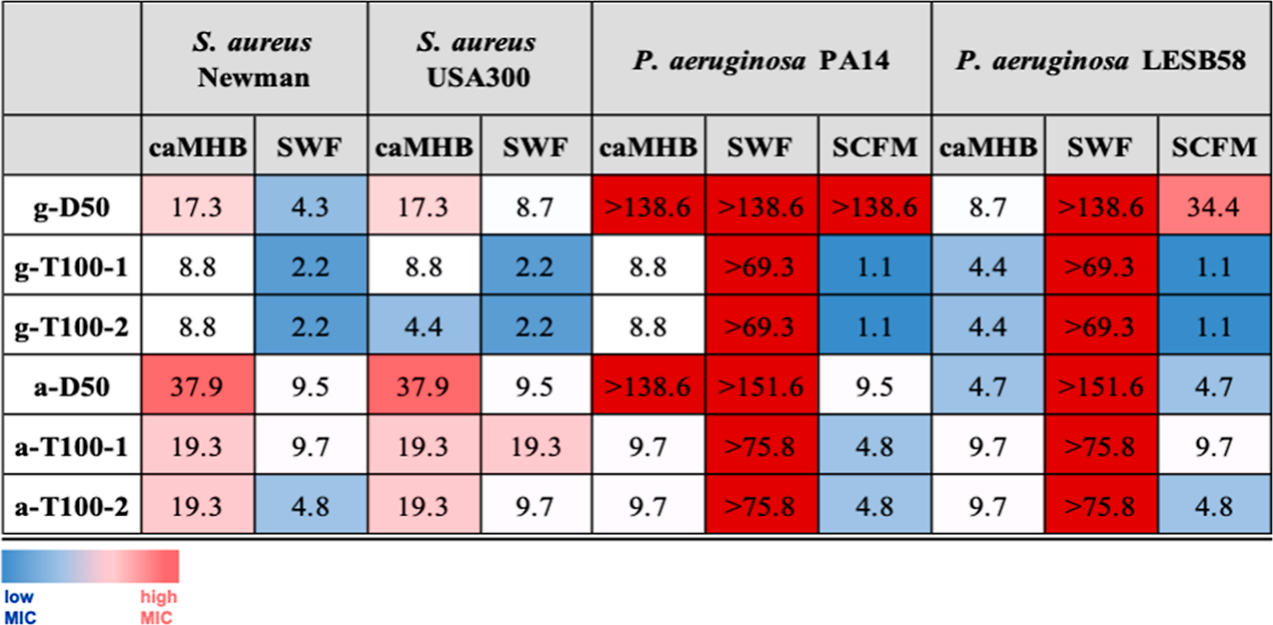
Antimicrobial Activity of the Polymers^a^

a MICs values expressed in μM of the copolymers tested in caMHB and SWF against *S. aureus* Newman and *S. aureus* USA300. MIC values expressed in μM tested in caMHB, SWF, and SCFM against *P. aeruginosa* PA14 and *P. aeruginosa* LESB58. The color gradient was used to highlight the most active compounds (clear blue) to the inactive compounds (dark red).

Subsequently, we tested the antimicrobial activity of the polymers in SWF against both *S. aureus* and *P. aeruginosa* and in SCFM against *P. aeruginosa*. *S. aureus* grew poorly *in vitro* using SCFM; therefore, it was excluded from the study. The results of our assay showed that for *S. aureus* strains, an enhanced activity of the copolymers was observed in the presence of SWF in comparison with standard caMHB medium (Table 1). For instance, a drastic change could be observed for a-D50 against *S. aureus* strains where the MIC in caMHB was 37.9 μM (256 μg mL^–1^), while in SWF, it was 9.5 μM (64 μg mL^–1^). For *P. aeruginosa*, in the case of the PA14 strain, the antimicrobial activity of the copolymers in SCFM was enhanced in comparison to caMHB. As can be observed, the activity of a-D50 was recovered in SCFM (Table 1). In the case of the clinical isolate, the same trend was observed except for g-D50, which was inactive in SCFM against both strains (Table 1). More surprisingly, the antimicrobial activity of all polymeric compounds was lost in SWF against *P. aeruginosa* strains (Table 1).

On the one hand, the improved antimicrobial activity of the polymers in SCFM against *P. aeruginosa* and in SWF against *S. aureus* compared to caMHB demonstrated that these materials held promise for use in certain therapeutic applications. On the other hand, the loss of activity against *Pseudomonas* in SWF was striking. A possible explanation could be an overexpression of efflux pumps by the bacteria in this environment, as this has been observed to occur in the presence of burn wound fluid.^44^ Efflux pumps are a resistance mechanism of bacteria that allows them to pump antibiotics out of the cell, minimizing damage. Another possibility might be the association of the polymers with proteins found in serum, which is present in SWF. On the one hand, protein binding is known to be an important factor affecting the activity and pharmacokinetic properties of antibiotics, with the protein-bound antibiotic fraction being considered inactive.^45^ On the other hand, the presence of serum components has been observed to result in a synergistic effect with certain compounds by facilitating their uptake by the bacteria.^46^ There is limited information on the effect of serum on antimicrobial polymers, although in a study of cationic methacrylate polymers, Thoma *et al*. observed enhanced activity against *S. aureus* in MHB-containing serum.^47^ This resembles our observations with SWF and *S. aureus*. Therefore, if there was an interaction with serum components, it seemed to affect the activity of the compounds in ways that differed between bacterial species, possibly due to differences in the bacterial surface.

**Figure 3:**
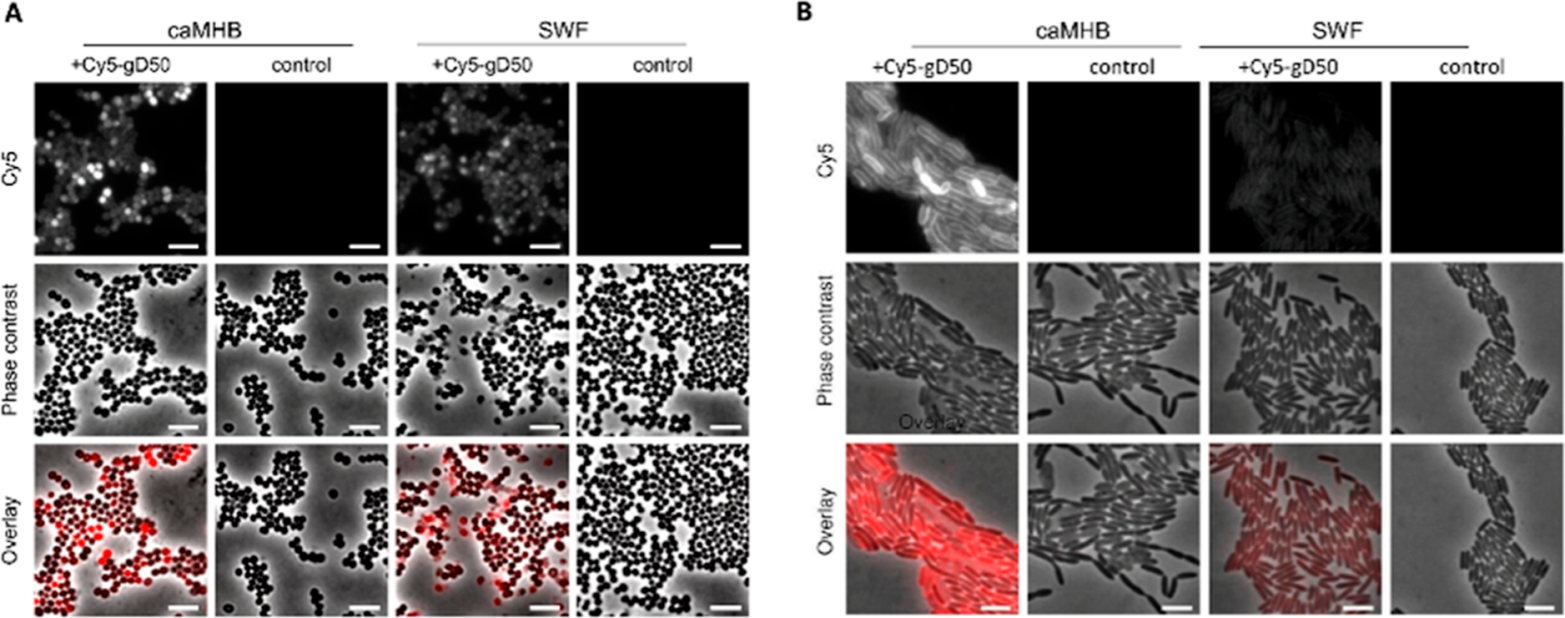
Fluorescence microscopy images of (A) *S. aureus* Newman and (B) *P. aeruginosa* LESB58 grown in either caMHB or SWF, after 30 min treatment with 64 μg/mL Cy5-g-D50 and fixation with paraformaldehyde. Control cultures were not treated with any compound. Scale bar: 4 μm.

In an attempt to understand the lack of activity against *P. aeruginosa* in SWF, we fluorescently labeled the compound g-D50 by attaching Cy5 to the RAFT agent (Figures S10 and S11). The MIC of the labeled compound was unaffected. We then treated both *S. aureus* Newman and *P. aeruginosa* LESB58 with the compound at a fixed concentration of 8.6 μM (64 μg mL^–1^), and observed the bacteria 30 min post-treatment, following fixation. As can be seen in Figure 3A, in contrast to *S. aureus*, where the compound associated with the bacteria in both media, there was very little interaction of the compound with *P. aeruginosa* in SWF (Figure 3B). It should be noted that the interaction of the compound with both bacteria was not homogeneous even in caMHB. This effect has been previously observed for both AMPs and synthetic mimics.^48,49^

Overall, our results demonstrate that the compound activity needs to be assessed in media that better mimic the infection environment, as it can vary widely under certain conditions. Similarly, the discrepancies seen in polymer activity against the two strains of *P. aeruginosa* illustrate the fact that there can be great differences between bacteria, even within the species level. Indeed, different strains are known to possess distinct LPS molecules or membrane lipid modifications.^50^ Consequently, there are certain risks and limitations when one chooses specific bacterial strains as “representative” organisms for antimicrobial activity testing.

### *In Vitro* Toxicity of Peptidomimetic Polymers in Mammalian Cells

The interaction of AMPs with cellular membranes has the potential to cause off-target effects and results in toxicity against mammalian cells.^51^ For instance, LL-37 is a human cathelicidin (AMPs) that induces apoptosis to epithelial cells.^52^ Additionally, some AMPs and polymer mimics have shown lytic activity against RBCs *in vitro*,^53^ and hydrophobicity is one of the parameters involved in their hemolytic behavior.^20^ We thus tested the copolymer family at concentrations up to 1 mg mL^–1^ at 37 °C against sheep RBCs (Figure S18). As can be observed in Table S5, none of the polymers caused considerable lysis of RBCs even at the highest concentration tested. For instance, even the most hydrophobic compounds, g-T100-2 and a-T100-2, showed less than 10% hemolysis at the highest concentration.

Subsequently, we studied the propensity of the polymers to cause RBC agglutination. As can be seen in Figure S19 and Table S5, the low-molecular-weight diblock copolymers (*i.e.,* g-D50 and a-D50) showed no hemagglutination at the highest concentration tested (138.5 and 151.6 μM, respectively, or 1024 μg mL^–1^). The most hydrophobic polymers g-T100-2 and a-T100-2 seemed to produce hemagglutination even at low concentrations (<1.1 μM or <16 μg mL^–1^, and 4.7 μM or 64 μg mL^–1^, respectively). The polymers characterized by an intermediate hydrophobic profile between the diblocks and the other triblocks, namely, g-T100-1 and a-T100-1, showed an intermediate hemagglutination effect (2.2 μM or 32 μg mL^–1^, and 9.7 μM 128 μg mL^–1^, respectively). In the case of the triblock copolymers, we noticed that the guanidinium copolymers caused more hemagglutination than the ammonium counterparts. However, hydrophobicity seemed to be the key parameter that modulated hemagglutination activity as the cationic percentage/proportion has been kept constant for all polymers.

**Figure 4:**
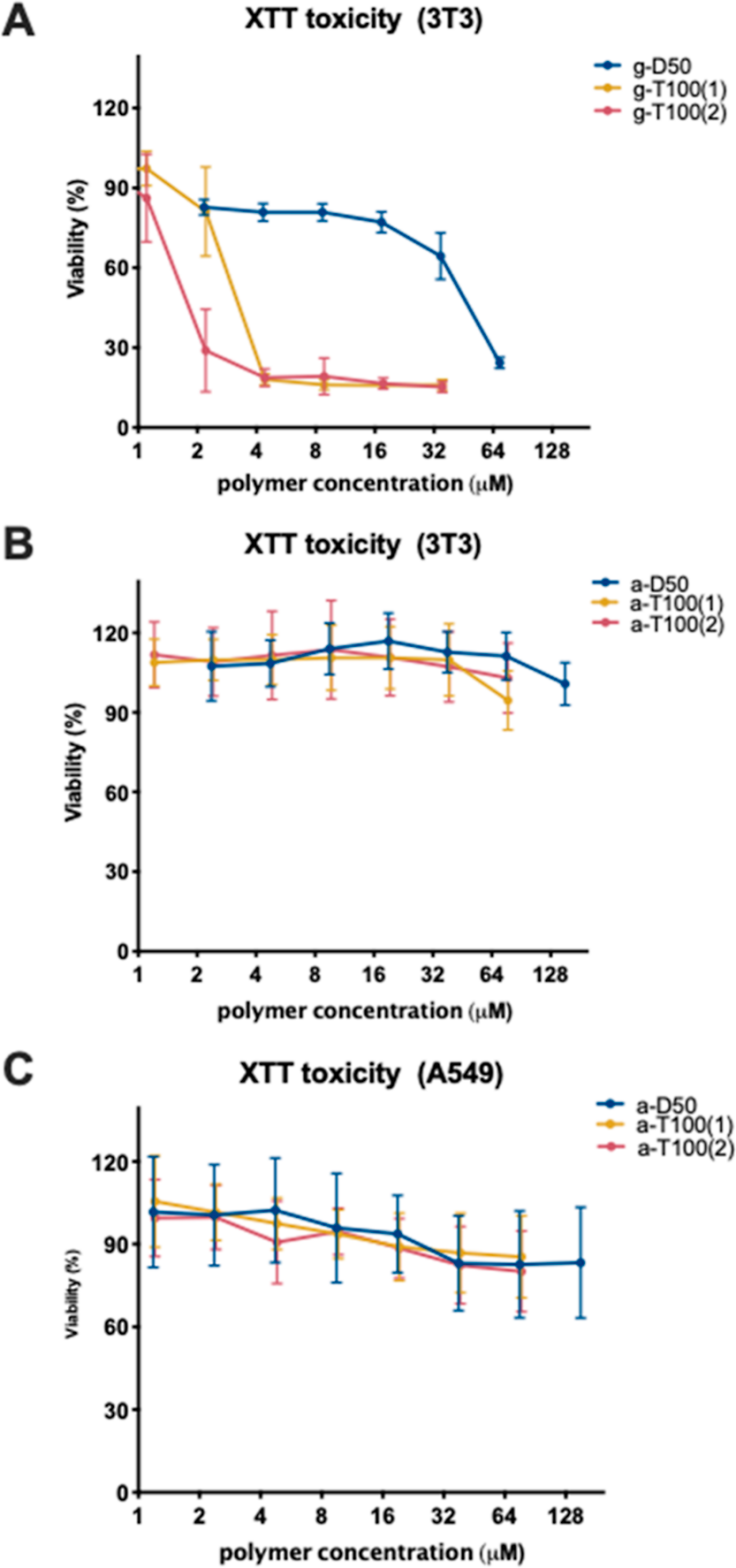
Cell viability in the presence of the polymers as measured by the reduction of XTT. (A) Guanidinium and (B) ammonium copolymers tested on the 3T3 cell line. (C) Ammonium copolymers tested on the A549 cell line. Shown are the averages of three biological replicates ± standard error.

*S. aureus* is one of the principal causes of recurrent infection in a chronic wound due to its ability to hinder wound healing.^32^ The antimicrobial activity of our polymers against *S. aureus* strains in SWF suggested that there was potential for application of the polymers as treatment in wound infections. As fibroblasts form an important component of connective tissue, it was pertinent to assess the cytotoxicity of the copolymers against the 3T3 murine cell fibroblast line, evaluated using an XTT assay. Ammonium polymers (a-D50, a-T100-1, and a-T100-2) did not show toxicity against the 3T3 cell line, as can be seen in Figure 4B. In this case, the monomer sequence and the molecular weight did not influence the toxicity of these polymers at the tested concentrations. However, the guanidinium polymers showed a higher cytotoxicity in comparison with their ammonium counterparts (Figure 4A), as observed previously.^23^ As can be seen in Figure 4A, the triblocks g-T100-1 and g-T100-2 exhibited high levels of cytotoxicity, while g-D50 was only toxic at the highest concentration tested. In this case, the molecular weight of the polymers was a crucial parameter that modulated the toxicity. In addition, the lower hydrophobicity profile of g-D50 compared to the triblock copolymers could be a reason for the reduction in toxicity in comparison with the more hydrophobic guanidinium triblock tested.

*P. aeruginosa* is the principal cause of death in CF patients due to lung infection.^33^ Since the polymers exhibited promising antimicrobial activity against *Pseudomonas* in SCFM, we investigated the cytotoxicity of the copolymers against human epithelial lung cells (A549). Since our study has shown that the charge group did not strongly influence the polymer activity against *Pseudomonas* and as the guanidinium copolymers have higher toxicity profiles, we focused on the most promising materials, the ammonium polymers. As can be seen in Figure 4C, the ammonium polymers do not exhibit a cytotoxic effect against A549 cells, even at the highest concentrations. IC_50_ values against 3T3 and A549 cells can be found in Table S6.

When designing new antimicrobial compounds, the goal is to optimize their therapeutic potential, so minimizing toxicity is as important as ensuring that they are active. On the one hand, our hemolysis and hemagglutination assays showed that hydrophobicity and molecular weight are defining factors for toxicity against RBCs. This is in line with the findings of Kuroda *et al*., who have shown a correlation between the higher hydrophobic content of statistical methacrylate copolymers with increased hemolysis.^20^ Additionally, the different results we obtained with the two triblock architectures are in agreement with the observations of Lie *et al*., who demonstrated that the segregation of the hydrophobic content played a crucial role in the toxicity of polymeric materials, as statistical copolymers were more hemolytic than diblock copolymers with similar molecular weight.^54^ Similarly, our group has previously shown a trend of increasing hemolytic behavior from diblocks to multiblocks to random copolymers, where the segregation of the hydrophobic content increased.^23^ On the other hand, toxicity against the 3T3 cell line was more influenced by the type of the positive charge with guanidinium copolymers showing higher levels of toxicity than their ammonium counterparts. Interestingly, the guanidinium polymers can be thought to mimic arginine-rich cell penetrating peptides (CCPs), which recently have been reported to induce membrane multilamellarity and fusion, allowing them to penetrate mammalian cells.^55^

### *In Vivo* Toxicity of Peptidomimetic Polymers Against *Galleria mellonella*

In recent years, not only invertebrates have emerged as a useful alternative to small mammals for *in vivo* assessments,^56^ due to the considerable similarities between the insect and mammalian innate immune system, but also there is increasing evidence that tests conducted in insects can provide good approximation and prediction for toxicity and pharmacokinetic properties in mammals.^57^ Specifically, the larvae of the greater wax moth *Galleria mellonella* have been used to study the relative toxicity of a variety of small molecules, showing a strong correlation with the toxicity profiles obtained against rats.^58^ Hence, we used *G. mellonella* larvae as an *in vivo* model to evaluate the cytotoxicity of our copolymers. In general, the data were in good agreement with the cytotoxicity assays against mammalian cells. Ammonium copolymers were not toxic to *G. mellonella* at any concentration tested. Triblock guanidinium copolymers showed the most toxic profiles, as larvae were killed at concentrations above 17.6 μΜ (256 μg mL^–1^). With regard to g-D50, the compound did not show toxicity to *G. mellonella*, even at the highest concentration tested (138.6 μM or 1024 μg mL^–1^). Survival plots at the different concentrations tested (Figure S20) can be found in the Supporting Information.

**Figure 5:**
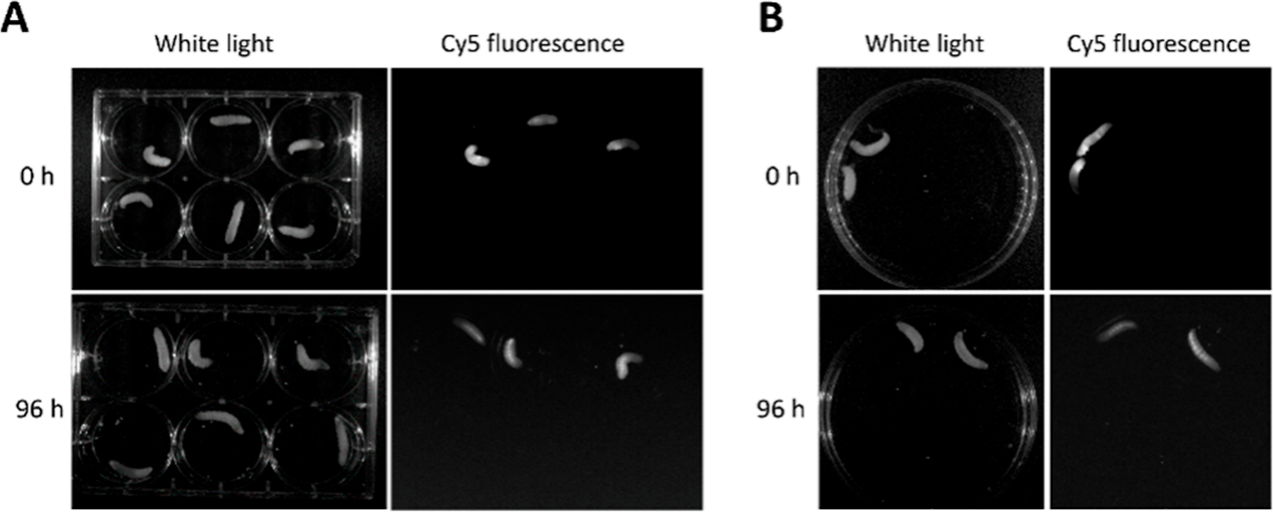
Fluorescence of Cy5-labeled g-D50 immediately following administration in *G. mellonella* larvae and 96 h later. (A) Administration of the compound was carried out by injection. The larvae shown at the top three wells were injected with the compound, while the remaining three larvae were injected with PBS. (B) Administration of the compound was carried out orally. The larvae were injected with PBS as the negative control for both experiments (imaging performed at the same time).

Since compound g-D50 was not toxic against *G. mellonella* larvae, we used the fluorescently labeled polymer Cy5-g-D50 to observe distribution and retention of the compounds in the larvae. Using both oral administration and injection, we observed that the compound quickly became well distributed in the body of the larvae and was mostly retained up to 96 h later, with a small amount excreted in the frass (Figure 5).

To the best of our knowledge, this is only the second study to use *G. mellonella* to assess the toxicity of polymeric compounds as it was recently used for polymeric nanocapsules.^59^ Our results showed that there is a good correlation between toxicity of polymers assessed against mammalian cell lines and using this insect model. A small discrepancy was seen with g-D50. However, it has been previously observed that *G. mellonella* can in fact be a better predictor for mammalian toxicity, while cell-based assays may overestimate the toxic effects of chemicals, especially those of low toxicity.^60^*In vivo* toxicity tests are very costly and involve the use of animals, posing ethical considerations. As such, they only happen in later stages of development when a lead compound is identified. The use of such invertebrate models has the advantage that large numbers can be used for experiments, allowing whole libraries of novel compounds to be tested at a very early stage. Finally, our observations following the administration of the fluorescently labeled compound to the larvae show that this model can be used to further investigate aspects, such as uptake and distribution of polymeric materials for an expanded range of applications.

### Studying the Interaction of the Polymers with Bacteria

To gain a better understanding of how the compounds work and whether they do mimic AMPs, we selected one compound for each bacterial strain to perform in-depth interaction studies. Guanidinium diblock copolymers have been reported to be able to target intracellular *S. aureus* infections.^23^ As a result, we selected the g-D50 copolymer from our family of copolymers as it showed relatively low MIC and a good biocompatibility against *G. mellonella* larvae. For *P. aeruginosa*, we further investigated the antimicrobial activity of ammonium copolymers as their MIC values were low in both caMHB and SCFM. The antimicrobial activity of the short diblock (a-D50) seemed to be dependent on the strain; therefore, we focused on the a-T100-1 copolymer (preliminary data suggested a stronger interaction with bacterial membranes than the a-T100-2 copolymer).

**Figure 6:**
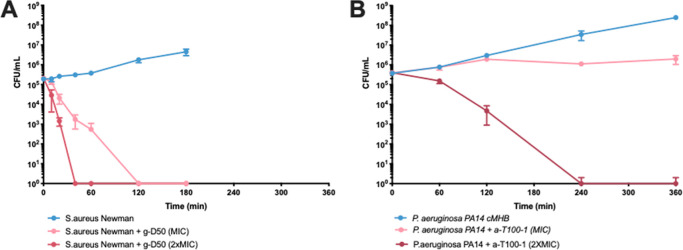
Time killing experiment of g-D50 against *S. aureus* Newman (A) and time killing experiments of a-T100-1 against *P. aeruginosa* PA14 (B) in caMHB. Control cultures were not treated with any compounds. Shown are the averages of three biological replicates ± standard error.

In order to establish how fast the compounds act, we performed time-killing kinetic assays of two lead compounds. In particular, we investigated the effect of exposure time and concentration of the polymer on bacterial viability. Over 99.9% of *S. aureus* Newman bacteria were killed by g-D50 after 40 min of exposure to 2 × MIC and after 2 h of exposure to the MIC concentration (Figure 6A). Over 99.9% of *P. aeruginosa* PA14 bacteria were killed after 4 h of exposure to 2 × MIC of a-T100-1. At the MIC, there was a single order of magnitude reduction in colony forming units rather than complete eradication of the bacteria (Figure 6B).

To investigate whether these lead compounds truly act as AMP mimics by disrupting the bacterial membrane or compromising the cell surface, we performed a series of assays using fluorescence and electron microscopy. As these experiments are conducted using higher bacterial densities than the MIC or time-killing assays, we first established the effect of 1 h exposure to different concentrations of the compounds to bacterial viability at a higher bacterial density of ∼10^8^ CFU mL^–1^ corresponding to an OD_600_ of 0.5. On the one hand, we observed that for *S. aureus* Newman, there was a single order of magnitude reduction in colony forming units after treatment with the MIC, with an additional 10-fold decrease for every doubling of the concentration of the compound (Figure S21A). On the other hand, for *P. aeruginosa*, at the MIC, there was only a twofold decrease in bacterial numbers and only a 10-fold decrease at 4 × MIC (Figure S21B). These results were confirmed by staining with the nucleic acid dye propidium iodide, which can only enter cells with compromised membranes. The cell-permeable SYTO-9 dye, which can enter intact cells, was used as a counter stain. At subinhibitory concentration, no uptake of propidium iodide could be observed either by *S. aureus* Newman treated with g-D50 or by *P. aeruginosa* PA14 treated with a-T100-1 (Figure S21C,D). At the MIC and at higher concentrations, uptake of propidium iodide correlated with the effect seen on bacterial viability. As a result, we were able to conduct further imaging studies on the effect of treatment using a concentration range close to the MIC, as that was expected to cause observable effects.

The effect of the compounds on the bacterial membrane was further observed by investigating how the treatment affected the staining of the lipophilic styryl dye FM 4-64 FX. The dye is almost nonfluorescent in aqueous solutions but becomes intensely fluorescent as it inserts into cell membranes. Therefore, loss of FM 4-64 FX staining would indicate complete destruction of the membrane. Bacteria treated with different concentrations of the compounds, or left untreated, were subsequently stained with the dye. As can be seen in Figure S22, at supraMIC concentrations, the staining of FM 4-64 FX was weaker than the control, indicating membrane disruption. However, the compounds did not act as surfactants to completely solubilize or “wash off” the membrane.

**Figure 7:**
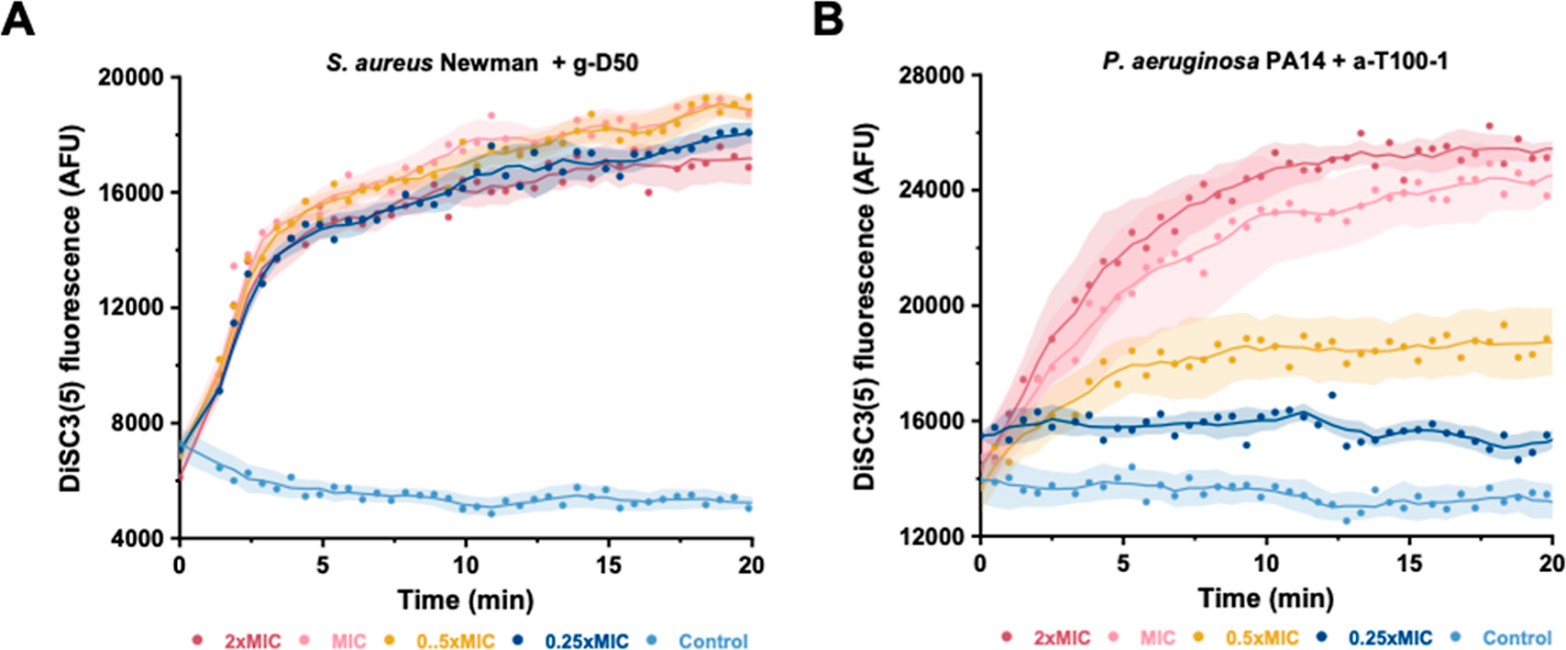
Membrane depolarization measured through the fluorescence of DiSC3(5) added to (A) *S. aureus* Newman, following treatment with either g-D50 at the indicated concentrations for g-D50, and (B) *P. aeruginosa* PA14 and following treatment with a-T100 at the indicated concentrations. Control cultures were not treated with any compounds. Shown are the averages of three biological replicates ± standard error.

Furthermore, we investigated the effect of the treatment on membrane ion permeability in *S. aureus* Newman and on inner membrane ion permeability of *P. aeruginosa* PA14 and subsequent membrane polarization. For this purpose, we used the potentiometric dye DiSC3(5), whose fluorescence becomes quenched as it enters energized cells. Membrane depolarization by treatment with compounds that dissipate membrane potential causes release of the dye and an increase in fluorescence. As can be observed in Figure 7, the addition of g-D50 to *S. aureus* Newman (Figure 7A) and a-T100-1 to *P. aeruginosa* PA14 (Figure 7B) caused rapid membrane depolarization even at concentrations below the MIC. In *P. aeruginosa* PA14, a concentration-dependent response could be observed (Figure 7B).

**Figure 8:**
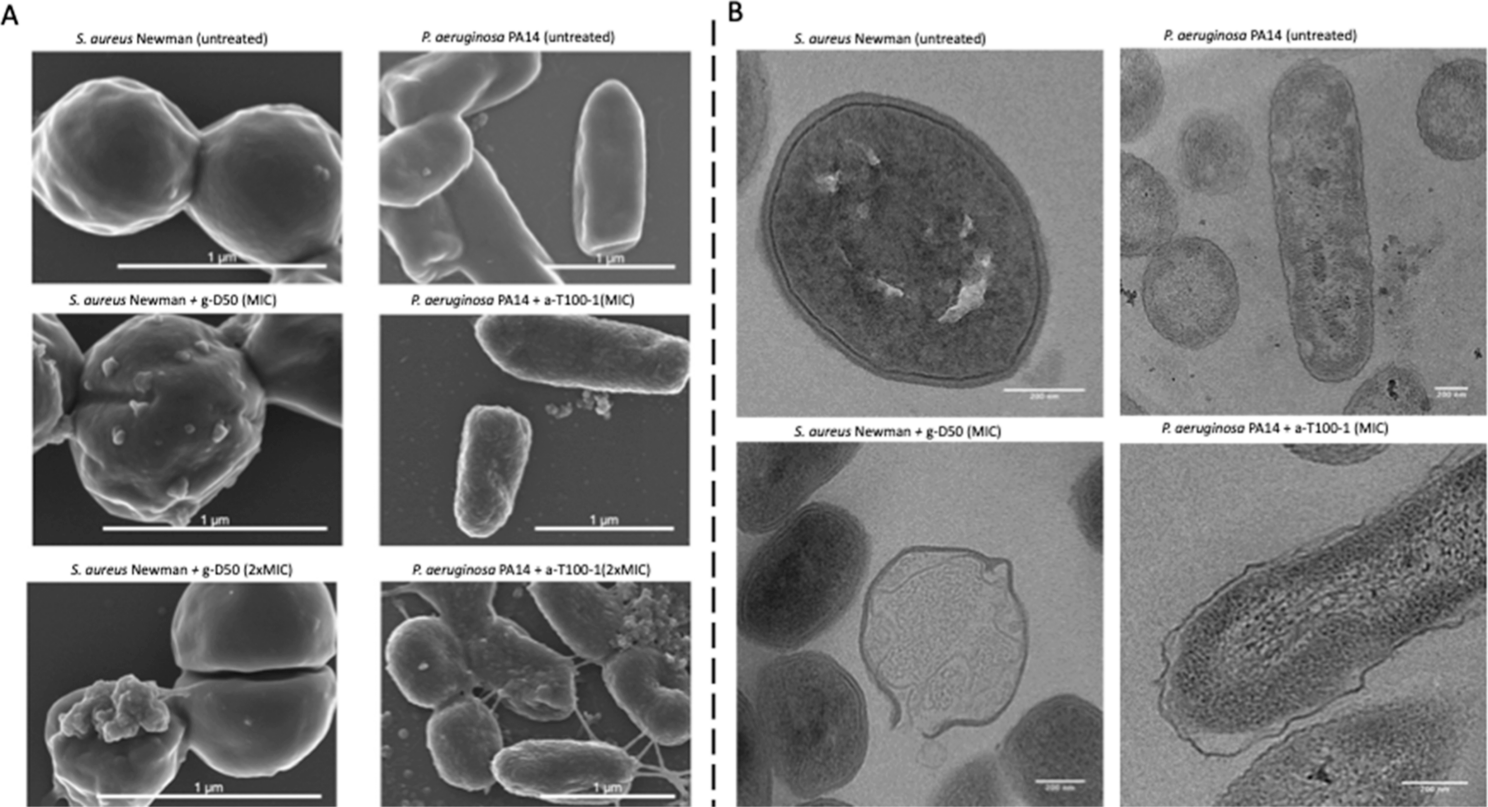
Representative electron micrographs. (A) Scanning electron micrographs of *S. aureus* Newman (untreated) and *S. aureus* Newman treated with g-D50 at MIC and 2× MIC concentration. Scanning electron micrographs of *P. aeruginosa* PA14 (untreated) and *P. aeruginosa* PA14 treated with a-T100-1 at MIC and 2× MIC. (B) Transmission electron micrographs of *S. aureus* Newman (untreated) and *S. aureus* Newman treated with g-D50 (MIC concentration). Transmission electron micrographs of *P. aeruginosa* PA14 (untreated) and *P. aeruginosa* PA14 treated with a-T100-1 (MIC concentration).

Membrane depolarization experiments indicated that the membrane of *S. aureus* and the inner membrane of *P. aeruginosa* were disrupted by the antimicrobial polymers in terms of their ion permeability. In order to investigate the effect of polymer treatment on the cell morphology, we used both scanning (SEM) and transmission (TEM) electron microscopy to image individual bacterial cells following polymer exposure, with untreated bacteria serving as controls. As seen above with the use of the fluorescently labeled g-D50, there was a certain degree of heterogeneity in the effect of the treatment of bacteria at the concentrations of polymers tested. Focusing on the bacteria that were morphologically affected, we could see through SEM that after 1 h of exposure to a-T100-1 at the MIC, the surfaces of *P. aeruginosa* PA14 cells lost their smoothness and developed superficial blebs (Figure 8A and S23, S24). The surface of *S. aureus* Newman treated with g-D50 at the MIC became roughened and covered by visible blebs (Figure 8A). Imaging thin-sectioned bacteria using TEM allowed for subcellular features to be unambiguously distinguished. In the case of *P. aeruginosa* PA14 cells exposed to a-T100-1 at the MIC for 1 h, TEM images revealed that in a subpopulation of bacteria, the outer membrane was visibly disrupted, with a larger periplasmic space between the inner and outer membrane in comparison with *P. aeruginosa* PA14 cells which have not been exposed to treatment (Figures 8B and S23, S24). In the case of *S. aureus* Newman treated with g-D50 at the MIC for 1 h (Figure 8B), lysed cells could be identified, where the cytoplasm of the lysed cells appeared substantially less electron dense in comparison with nonexposed cells, indicating the loss of intracellular material.

Here, our results using a variety of imaging techniques and membrane studies with the use of DiSC3(5) confirm that the compounds used in this study act as efficient AMP mimics with regard to their membrane disrupting abilities. Membrane depolarization experiments indicate potentiometric disruption to the inner membrane even at subinhibitory concentrations where there is no effect in bacterial viability or permeability to propidium iodide. This is a characteristic of the activity of certain AMPs, such as gramicidin, but is not universal to all AMPs. For example, polymyxin only causes minimal depolarization at concentrations much higher than the MIC.^61^ Additionally, our observations using electron microscopy confirmed the loss of structural integrity upon treatment. TEM imaging of *P. aeruginosa* was particularly revealing as the increased periplasm volume suggests that the osmotic integrity of the outer membrane has also been compromised. With regard to *S. aureus*, our findings are reminiscent of what was seen after treatment with AMPs (L12) at the MIC.^62^ Similarly, our observations using SEM are consistent with what has been reported for synthetic AMPs (PA-13) against *P. aeruginosa* cells, where significant changes in the bacterial membrane were observed, manifesting as roughening and corrugation.^63^

## Conclusions

It has been made clear in recent years that the activity of polymeric materials designed to mimic AMPs depends on factors, such as charge and hydrophobicity, and that some of these parameters can confer certain specificity toward Gram-positive or Gram-negative organisms. Here, we have demonstrated that in addition to these factors, the composition of the medium can greatly influence activity of synthetic AMPs, which could have important consequences for outcomes in a real infection scenario. Moreover, we illustrated the necessity to consider bacterial strain variability, which can also greatly affect compound activity. Finally, we demonstrated the usefulness of an invertebrate *in vivo* model in the early assessment of novel compound libraries for bioapplications.

## Experimental Section

### Materials

Acetonitrile for HPLC (>99.9%), diethyl ether, NIPAM (97%) dimethyl sulfoxide-*d*_6_ (DMSO, 99.5%), acryloyl chloride, 1,4-dioxane (≥99), chloroform (CHCl_3_), dichloromethane (DCM), ethylacetate (EtOAc), ethylenediamine, triethylamine (NEt3), trifluoro acetic acid (TFA), bis(*tert*-butoxycarbonyl)-2-methyl-2-thiopseudourea, magnesium sulfate (MgSO_4_), sodium bicarbonate (NaHCO_3_), hexane, ethyl acetate (EtOAc), *N,N*-diisopropylethylamine (DIPEA) Boc-anhydride, Dulbecco’s modified Eagle’s medium (DMEM), Müller-Hinton Broth type II (MHB cationic adjusted), phosphate saline solution (PBS) tablets, Roswell Park Memorial Institute medium (RPMI-1640), triton-X, concanavalin A from *Canavalia ensiformis* (Jack bean), and 3,3'-diropylthiadicarbocyanine iodine Disc3(5) were purchased from Sigma-Aldrich and used without further purification. DAPI (4',6-diamidino-2phenylindone), FM 4-64FX, fixable analog of FM 4-64 membrane stain, defibrinated sheep blood, fetal bovine serum (Gibco), hexamethyldisilazane (electronic grade, 99+%), LIVE/DEAD BacLight Bacterial Viability Kit for microscopy and quantitative assays (L7012), poly-D-lysine, and slowFade Gold Antifade Mountant were purchased from Fisher Scientific. Adhesion slides, coverslip (631-0123), round coverslip of 12 nm (631-1577P), and formaldehyde 4% aqueous solution buffered were purchased from VWR International Ldt (UK). *O*-(7-Aza-1*H*-benzotriazol-1-yl)-*N,N,N',N*'-tetramethyluronium hexafluorophosphate 99% (HATU) was purchased from Alfa Aesar. Cyanine5 amine was purchased from Lumiprobe GMHB. Pre-wetted RC tubings 1 kD were purchased from Spectrumlabs. XTT Cell proliferation Kit was purchased from ATCC. Glutaraldehyde solution 25% for electron microscopy was purchased from PanReac AppliChem. 2′-Azobis[2-(2-imidazolin-2-yl)propane]dihydrochloride (VA-044, Wako), Boc-anhydride (98%, Fluka), and sodium chloride (NaCl, Fisher-Scientific) were also used without any further purification steps. 2-((Butylthio)-carbonothioyl) thio propanoic acid (PABTC), 1,3-di-Boc-guanidinoethyl acrylamide (diBocGEAM), and *N*-*t*-butoxycarbonyl-1,2-diaminoethane (BocAEM) were synthesized and purified according to the reported literature.^64−66^

Buffered peptone water was purchased from Merck.

Galleria mellonela was purchased from Livefoods, UK.

The bacterial isolates used were *S. aureus* Newman (ATCC 25904), *S. aureus* USA300 Los Angeles County clone,^38^*P. aeruginosa* PA14 (standard laboratory strain),^39^ and the Liverpool epidemic strain *P. aeruginosa* LESB58.^40^ The cells lines used were embryonic fibroblast (*Mus musculus*) 3T3 (ATCC CRL-1658TM) and lung epithelial cells (*Homo sapiens*) A549 (ATCC CCL-185TM).

### Synthesis and Characterization

The synthesis of the monomers 1,3-di-Boc-guanidinoethyl acrylamide (diBoc-GEA) and Boc-AEAM, monomer characterization *via* NMR, block copolymerization, deprotection of polymers, and attachment of the Cy5 dye are described in the Supporting Information. Additionally, polymer characterization using NMR, size-exclusion chromatography, dynamic light scattering, HPLC, and SANS are also found in the Supporting Information.

## Methods

### Minimum Inhibitory Concentrations

Minimum inhibitory concentrations (MICs) were determined according to the standard Clinical Laboratory Standards Institute (CLSI) broth microdilution method (M07-A9-2012)^67^ using cation-adjusted Müller-Hinton broth (caMHB). Additionally, MICs were determined using SWF (peptone water/fetal bovine serum 50:50 %v/v) as described by Werthén *et al*.^36^ and SCFM as described by Palmer *et al*.^34^ A single colony of bacteria grown on LB agar plates was picked and resuspended in fresh medium. The concentration of bacterial cells was adjusted by measuring the optical density at 600 nm (OD_600_) to obtain an equivalent to a McFarland standard of 0.5 (OD_600_ ∼ 0.08–0.1), corresponding to a bacterial concentration of ∼10^8^ colony forming units per mL (CFU mL^–1^). The solution was diluted 100-fold to obtain a concentration of 10^6^ CFU mL^–1^. Polymers were dissolved in the respective media, and 50 μL of each polymer solution was added to the wells of Corning Costar TC-treated 96-well plates followed by the addition of the same volume of bacterial suspension, resulting in a final bacterial density of 5 × 10^5^ CFU mL^–1^. The polymer concentrations tested ranged from 512 to 8 μg mL^–1^, in a twofold dilution series. The microwell plates were incubated at 37 °C for 18 h, and growth was evaluated by eye. Triplicates were performed for each concentration. Triplicates with just bacteria and triplicates with media only were used as positive and negative control, respectively. The experiment was repeated three times, and the highest value obtained was reported. MIC values were reported in μM due to the difference in the molecular weight of the polymeric material and to enable comparison of the antimicrobial activity with small-molecule antibiotics.

### Bacterial Killing Experiments

For the time-killing assays, a bacterial suspension of 10^6^ CFU mL^–1^ was prepared, from exponentially growing cells (bacterial density similar to that used in the MIC experiments). This bacterial suspension was placed into screw cap test tubes of 5 mL (LabDirect) and incubated in the presence of polymeric compounds at the MIC and 2× MIC in a final volume of 2 mL at 37 °C with shaking conditions. Bacteria were counted by taking 50 μL aliquots at regular time intervals (*t* = 0, 20, 40, 60, 120, and 280 min for *S. aureus* Newman and *t* = 0, 60, 120, 280, 360, and 420 min for *P. aeruginosa* PA14), making serial dilutions and plating on LB agar. For assaying killing at high bacterial densities, bacteria were grown in caMHB until OD_600_ reached 0.5 (corresponding to ∼10^8^ CFU mL^–1^). Bacteria were aliquoted in 2 mL tubes, and the lead compounds were added at the indicated final concentrations (0.5× MIC, MIC, 2× MIC, and 4× MIC). The bacteria were incubated in the presence of the compound for 1 h at 37 °C, and then, the cultures were serially diluted in PBS. Aliquots were plated on LB agar. Three independent experiments were performed, and the average and standard deviation were reported.

### Hemolysis Assay

Sheep red blood cells (RBCs) were washed with PBS *via* centrifugation (4500*g* for 1 min) at least three times, until the supernatant was clear. Polymers were dissolved in PBS and serially diluted (1024 to 16 μg mL^–1^). A solution of 2% (v/v) Triton X-100 was used as a positive control, and a solution of PBS was used as a negative control. Each treatment and controls were performed in triplicates. RBCs (100 μL, 6% (v/v)) in PBS were added to each well of Corning Costar TC-treated 96-well plates. Then, 100 μL of each treatment was added and was mixed before being incubated at 37 °C for 3 h. The 96-well plates were centrifuged at 600*g* for 10 min, and 100 μL of the supernatant was transferred to a new plate. The absorbance at 540 nm was measured using a Cytation 3 microplate reader (BioTek). Three independent experiments were performed.

### Hemagglutination Assay

Hemagglutination assay was performed according to the protocol established by Banerjee *et al*.^68,69^ Sheep RBCs were washed with PBS *via* centrifugation (4500*g* for 1 min) at least three times, until the supernatant was clear. RBCs were diluted in PBS buffer, and 50 μL was transferred to each well of Corning 96-well clear round-bottom TC-treated microplates. Polymers were dissolved in PBS and serially diluted. Then, 50 μL aliquots of the polymer solutions were transferred to the wells containing the RBCs, using triplicate wells for each concentration. The resulting polymer concentrations tested ranged from 16 to 1024 μg mL^–1^. Concanavalin A (50 mg mL^–1^) solution was used as a positive control, and PBS was used as a negative control. The microplate was incubated at 37 °C for 1 h. Three independent experiments were performed. The highest hemagglutination values were reported (the data was reported in μM due to the difference in molecular weight of the polymeric material and in order to enable comparison).

### *In Vitro* Toxicity of Antimicrobial Polymers in Eukaryotic Cells

Cells were seeded into Corning Costar TC-treated 96-well plates at a density of 2 × 10^4^ cells per well and cultured at 37 °C in basal medium DMEM with 10% fetal calf serum (for 3T3 cells) or 10% fetal bovine serum (for A549 cells) supplemented with 1% (v/v) glutamine and 1% (v/v) penicillin/streptomycin. The cells were allowed to grow for 24 h. The medium was then replaced with fresh medium and complemented with solutions of polymers (ranging from 16 up to 1024 μg mL^–1^). Cells were further incubated for 24 h. The media were replaced, and the cells were washed twice with PBS and then fresh medium containing 2,3-bis-(2-methoxy-4-nitro-5-sulfophenyl)-2H-tetrazolium-5-carboxanilide) (XTT) at 0.2 mg mL^–1^, and *N*-methyl dibenzopyrazine methyl sulfate (250 μM) was added and incubated for 18 h. Cells were then transferred to a plate reader, and absorbance at 450 and 650 nm was measured. Each polymeric treatment was performed in triplicates, and three independent experiments were performed.

### *G. mellonella* Assays

*G. mellonella* was used to assess the toxicity of polymeric compounds *in vivo* as previously reported.^70^ The fifth instar larvae were maintained in sawdust at 4–8 °C (1–3 days). Larvae were weighed, and only those weighing 250 mg ± 10 mg were used in the experiment to ensure that the correct polymeric dose was injected. Eight larvae were used for each dose of the polymeric compound tested. Larvae were placed on ice for immobilization during injections. Before the injection, the surface of the larvae was sterilized using 70% ethanol. The polymeric solution (10 μL, ranging from 256 to 1024 μg mL^–1^ as the final concentration) was injected using a Hamilton syringe in the proleg. PBS was used as a negative control. The larvae were placed in Petri dishes in the dark at room temperature, and the survival was monitored for 7 days.

For the study of distribution and retention of the fluorescently labeled polymer, polymeric solution was prepared in PBS and administered to the larvae either by injection, as described above, or orally by force-feeding. The final concentration of the compound was 128 μg mL^–1^ of larval weight. To assist with oral administration, the polymer solution was aspirated using a 20 μL pipette and gel loading tips were used. The larvae were imaged using a Syngene G:BOX XR5 system equipped with an Epi red LED (640 nm) to allow for Cy5 excitation.

### Fluorescence Microscopy

Bacteria were grown in caMHB, unless otherwise stated, to the mid-exponential phase until an OD_600_ of ∼0.5 was reached.

To investigate binding of the fluorescent polymer, 500 μL aliquots of bacteria grown in caMHB or SWF were transferred to 2 mL centrifuge tubes and either treated with a final concentration of 64 μg mL^–1^ Cy5-gD50 or left untreated. The suspensions were incubated at 37 °C, shaking at 700 rpm for 30 min. They were then harvested by centrifugation (10,000 rpm, 5 min), washed with PBS, and fixed using 4% formaldehyde in PBS for 10 min. They were again washed with PBS, and 3 μL was deposited on slides containing agar pads and allowed to air-dry. Then, SlowFade gold antifade mountant was added, and a coverslip was mounted.

To investigate the effect of the compounds on membrane staining, 400 μL aliquots of bacteria were transferred to 2 mL tubes and incubated in the presence of the lead compounds at the desired concentrations for 1 h. 10 min prior to the end of the incubation period, the lipophilic dye FM 4-64 FX was added. The bacteria were then washed in PBS and fixed using 4% formaldehyde in PBS for 10 min. They were again washed with PBS and applied to agar-pad slides for microscopy as described above.

For staining with SYTO 9 and propidium iodide, the LIVE/DEAD BacLight bacterial viability kit (L7007) from Invitrogen was used according to the manufacturer’s instructions. Briefly, exponentially growing cells were aliquoted into 2 mL tubes and treated with the indicated concentration of gD50 for *S. aureus* Newman or a-T100-1 for *P. aeruginosa* PA14 for 1 h. They were then washed twice and resuspended in 0.9% w/v NaCl. LIVE/DEAD solution consisting of equal volumes of component A and component B was then added at 3 μL mL^–1^ bacterial suspension so that the final concentration of SYTO 9 was 5 μM and of propidium iodide was 30 μM. The bacteria were then applied to agar pad slides and visualized by microscopy. Imaging was performed on a Leica DMi8 widefield fluorescence microscope, equipped with a Hamamatsu Orca Flash 4.0 V2 camera. The 100× oil objective was used together with the FITC and TXR filters.

### Inner Membrane Depolarization Assay

Overnight cultures of *S. aureus* Newman and *P. aeruginosa* PA14 were diluted in caMHB and grown to mid-exponential. They were then washed in a 5 mM HEPES buffer containing 20 mM glucose, pH = 7.3 (HEPES–glucose buffer). For *S. aureus* Newman, the culture was adjusted to an OD_600_ of 0.35, and 85 μL aliquots were transferred to a black flat-bottomed μClear 96-well plate (Greiner). Then, 5 μL of DiSC3(5) dye was added from a stock solution of 40 μM in HEPES–glucose buffer containing 20% DMSO, giving a final concentration of the dye of 2 μM. Following uptake of the dye, 5 μL of KCl was added to a final concentration of 100 mM, followed by 5 μL of gD50 from stock solutions at 20×, the desired final concentrations. For *P. aeruginosa* PA14, permeabilization of the outer membrane of the bacteria is necessary to allow uptake of the dye, so after washing the bacteria in HEPES–glucose buffer, EDTA was added at a final concentration of 0.5 mM. Bacterial density was adjusted to an OD_600_ of 0.2, and the experiment was conducted as mentioned above with compound a-T100-1 and using a final concentration of DiSC3(5) of 2.5 μM. Fluorescence was measured using a BMG LABTECH OPTIMA plate reader with the plate shaken between measurements. Three independent experiments were perfomed, and the average and standard deviation were reported.

### Scanning Electron Microscopy

From an overnight of bacterial suspension in caMHB, a fresh inoculum was prepared and incubated at 37 °C with shaking until the mid-exponential phase was obtained (10^7–8^ CFU mL^–1^ in caMHB). This bacterial solution was incubated in the presence of polymeric compounds (at the MIC and 2× MIC) at 37 °C for 1 h. Then, the cells were pelleted by centrifugation at 6000*g* for 1 min, followed by three washes in PBS. Circular glass cover slips (12 mm diameter) were incubated with 50 μL of poly-lysine in a 24-well tissue culture plate. After 15 min, the poly-lysine solution was removed, and the cover slips were left to dry. The bacterial cell pellets were resuspended in 400 μL of PBS, and 100 μL was added to the cover slips. After 30 min of incubation, the excess volume was removed. The cells were then fixed at 4 °C overnight with a 2.5% glutaraldehyde solution in PBS. After fixation, the 2.5% glutaraldehyde solution was discarded, and the cover slips where rinsed three times with PBS. The coverslips were transferred to clean wells, and dehydration was performed using an ethanol gradient (from 20, 50, 70, 90, 100, and 100%) for 10 min at each concentration. After complete dehydration, the cover slips were moved to clean wells and were incubated with 0.5 mL of hexamethyldisilazane (HDMS) as a drying agent for 30 min. The HDMS solution was then discarded, and the cover slips were moved to clean wells and left to dry in a laminar flow cabinet for 30 min. Copper tape was added to SEM sample holders, and the cover slips where placed on top. Finally, the samples were sputtered using a carbon coater (Emitech K950X). Imaging was performed at the Warwick Electron Microscopy Research Technology Platform on a Zeiss Gemini scanning electron microscope equipped with an in-lens detector, at a voltage of 1 kV.

### Transmission Electron Microscopy

From an overnight of bacterial culture in caMHB, a fresh inoculum was prepared and incubated at 37 °C in shaking until the mid-exponential phase was obtained (∼10^8^ CFU mL^–1^ in caMHB). This bacterial solution was incubated in the presence of polymeric compounds at the MIC, at 37 °C for 1 h. Then, the cells were pelleted by centrifugation at 6000*g* for 1 min, followed by three washes in PBS. The cells were then fixed at 1 h at room temperature with a 2.5% glutaraldehyde solution in PBS. After fixation, the 2.5% glutaraldehyde solution was discarded, and the pellets where rinsed three times with PBS. Cells were subsequently incubated with 1% osmium tetroxide for 60 min at room temperature. Following washing with PBS, the bacterial samples were dehydrated in a graded acetone series and transferred to graded acetone–epoxy resin mixtures for 45 min each until pure resin incubated overnight at a constant temperature. Finally, the specimens were sectioned with an ultramicrotome. Imaging was performed at the Warwick Advanced Bioimaging Research Technology Platform on a JEOL 2100Plus LaB6 transmission electron microscope equipped with a Gatan OneView IS camera.

## Supporting Information Available

Additional experimental details of the monomer synthesis, polymer synthesis conditions, Boc-deprotection, Cy5 conjugation, and SANs experiments, NMR spectra of the monomers, polymer chain extensions, and Boc-deprotection, SEC measurement, DLS measurements, UV–vis measurements, MIC values, hemagglutination values, and fluorescent microscopy images of bacteria exposed to polymeric material visualized with different dyes (PDF)

## Abbreviations

ESKAPE: *Enterococcus faecium*, *Staphylococcus aureus*, *Klebsiella pneumoniae*, *Acinetobacter baumannii*, *Pseudomonas aeruginosa*, and *Enterobacter* spp.
AMR: antimicrobial resistance
AMPs: antimicrobial peptides
sAMPs: synthetic antimicrobial peptides
RAFT: reversible addition–fragmentation chain-transfer polymerization
MIC: minimum inhibitory concentration
CF: cystic fibrosis
SWF: synthetic wound fluid
SCFM: synthetic cystic fibrosis sputum medium
GEAM: (guanidino-ethyl)acrylamide
AEAM: *N*-(2-aminoethyl)acrylamide
NIPAM: *N*-isopropylacrylamide
DP: degree of polymerization
D̵: polydispersity
SEC: size-exclusion chromatography
HPLC: high-performance liquid chromatography
SANS: small-angle neutron scattering
PBS: phosphate-buffered saline
DLS: dynamic light scattering
LCST: lower critical solution temperature
*S. aureus*: *Staphylococcus aureus*
*P. aeruginosa*: *Pseudomonas aeruginosa*
caMHB: cationic adjusted Müller-Hinton broth type II
LPS: lipopolysaccharide
RBCs: red blood cells
IC50: 50% cell viability
CCPs: cell penetrating peptides
SEM: scanning electron microscopy
TEM: transmission electron microscopy

## References

[ref1] Antimicrobial resistance. World Health Organization (WHO), 2021. https://www.who.int/news-room/fact-sheets/detail/antimicrobial-resistance (accessed 10 Nov, 2021).

[ref2] Murray C. J.; Ikuta K. S.; Sharara F.; Swetschinski L.; Aguilar G. R.; Gray A.; Han C.; Bisignano C.; Rao P.; Wool E. Global burden of bacterial antimicrobial resistance in 2019: a systematic analysis. Lancet 2022, 339, 629–655. 10.1016/s0140-6736(21)02724-0. PMC8841637 35065702

[ref3] Boucher H. W.; Talbot G. H.; Bradley J. S.; Edwards J. E.; Gilbert D.; Rice L. B.; Scheld M.; Spellberg B.; Bartlett J. Bad Bugs, No Drugs: No ESKAPE! An Update from the Infectious Diseases Society of America. Clin. Infect. Dis. 2009, 48, 1–12. 10.1086/595011. 19035777

[ref4] Butler M. S.; Blaskovich M. A.; Cooper M. A. Antibiotics in the clinical pipeline at the end of 2015. J. Antibiot. 2017, 70, 3–24. 10.1038/ja.2016.72. 27353164

[ref5] Zhang L.-j.; Gallo R. L. Antimicrobial peptides. Curr. Biol. 2016, 26, R14–R19. 10.1016/j.cub.2015.11.017. 26766224

[ref6] Mookherjee N.; Anderson M. A.; Haagsman H. P.; Davidson D. J. Antimicrobial host defence peptides: functions and clinical potential. Nat. Rev. Drug Discov. 2020, 19, 311–332. 10.1038/s41573-019-0058-8. 32107480

[ref7] Li J.; Koh J. J.; Liu S.; Lakshminarayanan R.; Verma C. S.; Beuerman R. W. Membrane active antimicrobial peptides: Translating mechanistic insights to design. Front. Neurosci. 2017, 11, 73 10.3389/fnins.2017.00073. 28261050 PMC5306396

[ref8] Benfield A. H.; Henriques S. T. Mode-of-Action of Antimicrobial Peptides: Membrane Disruption vs. Intracellular Mechanisms. Front. Med. Technol. 2020, 2, 610997 10.3389/fmedt.2020.610997. 35047892 PMC8757789

[ref9] Jangir P. K.; Ogunlana L.; MacLean R. C. Evolutionary constraints on the acquisition of antimicrobial peptide resistance in bacterial pathogens. Trends Microbiol. 2021, 29, 1058–1061. 10.1016/j.tim.2021.03.007. 33836929

[ref10] Bacalum M.; Radu M. Cationic antimicrobial peptides cytotoxicity on mammalian cells: An analysis using therapeutic index integrative concept. Int. J. Pept. Res. Ther. 2015, 21, 47–55. 10.1007/s10989-014-9430-z.

[ref11] Otvos L.; Wade J. D. Current challenges in peptide-based drug discovery. Front. Chem. 2014, 2, 62 10.3389/fchem.2014.00062. 25152873 PMC4126357

[ref12] Bray B. L. Large-scale manufacture of peptide therapeutics by chemical synthesis. Nat. Rev. Drug Discovery 2003, 2, 587–593. 10.1038/nrd1133. 12815383

[ref13] Jenei S.; Tiricz H.; Szolomájer J.; Tímár E.; Klement É.; Al Bouni M. A.; Lima R. M.; Kata D.; Harmati M.; Buzás K.; et al. Potent Chimeric Antimicrobial Derivatives of the Medicago truncatula NCR247 Symbiotic Peptide. Front. Microbiol. 2020, 11, 270 Original Research 10.3389/fmicb.2020.00270. 32153547 PMC7047876

[ref14] Irazazabal L. N.; Porto W. F.; Ribeiro S. M.; Casale S.; Humblot V.; Ladram A.; Franco O. L. Selective amino acid substitution reduces cytotoxicity of the antimicrobial peptide mastoparan. Biochim. Biophys. Acta 2016, 1858, 2699–2708. 10.1016/j.bbamem.2016.07.001. 27423268

[ref15] Moad G.; Chiefari J.; Chong Y. K.; Krstina J.; Mayadunne R. T. A.; Postma A.; Rizzardo E.; Thang S. H. Living free radical polymerization with reversible addition - fragmentation chain transfer (the life of RAFT). Polym. Int. 2000, 49, 993–1001. 10.1002/1097-0126(200009)49:9<993::aid-pi506>3.0.co;2-6.

[ref16] Gody G.; Maschmeyer T.; Zetterlund P. B.; Perrier S. Pushing the limit of the RAFT process: Multiblock copolymers by one-pot rapid multiple chain extensions at full monomer conversion. Macromolecules 2014, 47, 3451–3460. 10.1021/ma402435n.

[ref17] Hu J.; Qiao R.; Whittaker M. R.; Quinn J. F.; Davis T. P. Synthesis of star polymers by RAFT polymerization as versatile nanoparticles for biomedical applications. Aust. J. Chem. 2017, 70, 1161–1170. 10.1071/ch17391.

[ref18] Zhang J.; Gody G.; Hartlieb M.; Catrouillet S.; Moffat J.; Perrier S. Synthesis of Sequence-Controlled Multiblock Single Chain Nanoparticles by a Stepwise Folding-Chain Extension-Folding Process. Macromolecules 2016, 49, 8933–8942. 10.1021/acs.macromol.6b01962.

[ref19] Kerr A.; Hartlieb M.; Sanchis J.; Smith T.; Perrier S. Complex multiblock bottle-brush architectures by RAFT polymerization. Chem. Commun. 2017, 53, 11901–11904. 10.1039/c7cc07241d. 29043301

[ref20] Mortazavian H.; Foster L. L.; Bhat R.; Patel S.; Kuroda K. Decoupling the Functional Roles of Cationic and Hydrophobic Groups in the Antimicrobial and Hemolytic Activities of Methacrylate Random Copolymers. Biomacromolecules 2018, 19, 4370–4378. 10.1021/acs.biomac.8b01256. 30350596 PMC6238640

[ref21] Kuroki A.; Sangwan P.; Qu Y.; Peltier R.; Sanchez-Cano C.; Moat J.; Dowson C. G.; Williams E. G. L.; Locock K. E. S.; Hartlieb M.; et al. Sequence Control as a Powerful Tool for Improving the Selectivity of Antimicrobial Polymers. ACS Appl. Mater. Interfaces 2017, 9, 40117–40126. 10.1021/acsami.7b14996. 29068226

[ref22] Palermo E. F.; Lienkamp K.; Gillies E. R.; Ragogna P. J. Antibacterial Activity of Polymers: Discussions on the Nature of Amphiphilic Balance. Angew. Chem., Int. Ed. 2019, 58, 3690–3693. 10.1002/anie.201813810. 30653795

[ref23] Kuroki A.; Kengmo Tchoupa A.; Hartlieb M.; Peltier R.; Locock K. E. S.; Unnikrishnan M.; Perrier S. Targeting intracellular, multi-drug resistant Staphylococcus aureus with guanidinium polymers by elucidating the structure-activity relationship. Biomaterials 2019, 217, 119249 10.1016/j.biomaterials.2019.119249. 31279102

[ref24] Ersoy S. C.; Heithoff D. M.; Barnes L.; Tripp G. K.; House J. K.; Marth J. D.; Smith J. W.; Mahan M. J. Correcting a Fundamental Flaw in the Paradigm for Antimicrobial Susceptibility Testing. EBioMedicine 2017, 20, 173–181. 10.1016/j.ebiom.2017.05.026. 28579300 PMC5478264

[ref25] Lapierre S. G.; Phelippeau M.; Hakimi C.; Didier Q.; Reynaud-Gaubert M.; Dubus J. C.; Drancourt M. Cystic fibrosis respiratory tract salt concentration: An exploratory cohort study. Medicine 2017, 96, e8423 10.1097/md.0000000000008423. 29381919 PMC5708918

[ref26] Dürr U. H. N.; Sudheendra U. S.; Ramamoorthy A. LL-37, the only human member of the cathelicidin family of antimicrobial peptides. Biochim. Biophys. Acta Biomembr. 2006, 1758, 1408–1425. 10.1016/j.bbamem.2006.03.030. 16716248

[ref27] Walkenhorst W. F. Using adjuvants and environmental factors to modulate the activity of antimicrobial peptides. Biochim. Biophys. Acta Biomembr. 2016, 1858, 926–935. 10.1016/j.bbamem.2015.12.034. 26751595

[ref28] Smart M.; Liu W.-K.; Ha B.-Y. Opposing effects of cationic antimicrobial peptides and divalent cations on bacterial lipopolysaccharides. Phys. Rev. E: Stat., Nonlinear, Soft Matter Phys. 2017, 96, 042405 10.1103/physreve.96.042405. 29347628

[ref29] Mercer D. K.; Torres M. D. T.; Duay S. S.; Lovie E.; Simpson L.; von Köckritz-Blickwede M.; de la Fuente-Nunez C.; O’Neil D. A.; Angeles-Boza A. M. Antimicrobial Susceptibility Testing of Antimicrobial Peptides to Better Predict Efficacy. Front. Cell. Infect. Microbiol. 2020, 10, 326 10.3389/fcimb.2020.00326. 32733816 PMC7358464

[ref30] Zhang Y.; Cai J.; Li C.; Wei J.; Liu Z.; Xue W. Effects of thermosensitive poly(N-isopropylacrylamide) on blood coagulation. J. Mater. Chem. B 2016, 4, 3733–3749. 10.1039/c6tb00823b. 32263312

[ref31] Jain K.; Vedarajan R.; Watanabe M.; Ishikiriyama M.; Matsumi N. Tunable LCST behavior of poly(N-isopropylacrylamide/ionic liquid) copolymers. Polym. Chem. 2015, 6, 6819–6825. 10.1039/c5py00998g.

[ref32] Trivedi U.; Parameswaran S.; Armstrong A.; Burgueno-Vega D.; Griswold J.; Dissanaike S.; Rumbaugh K. P. Prevalence of Multiple Antibiotic Resistant Infections in Diabetic versus Nondiabetic Wounds. J. Pathog. 2014, 2014, 1–6. 10.1155/2014/173053. PMC4099163 25054067

[ref33] Bhagirath A. Y.; Li Y.; Somayajula D.; Dadashi M.; Badr S.; Duan K. Cystic fibrosis lung environment and Pseudomonas aeruginosa infection. BMC Pulm. Med. 2016, 16, 174 10.1186/s12890-016-0339-5. 27919253 PMC5139081

[ref34] Palmer K. L.; Aye L. M.; Whiteley M. Nutritional cues control Pseudomonas aeruginosa multicellular behavior in cystic fibrosis sputum. J. Bacteriol. 2007, 189, 8079–8087. 10.1128/jb.01138-07. 17873029 PMC2168676

[ref35] Deschamps E.; Schaumann A.; Schmitz-Afonso I.; Afonso C.; Dé E.; Loutelier-Bourhis C.; Alexandre S. Membrane phospholipid composition of Pseudomonas aeruginosa grown in a cystic fibrosis mucus-mimicking medium. Biochim. Biophys. Acta Biomembr. 2021, 1863, 183482 10.1016/j.bbamem.2020.183482. 33002450

[ref36] Werthén M.; Henriksson L.; Jensen P. Ø.; Sternberg C.; Givskov M.; Bjarnsholt T. An in vitro model of bacterial infections in wounds and other soft tissues. APMIS 2010, 118, 156–164. 10.1111/j.1600-0463.2009.02580.x. 20132180

[ref37] Baba T.; Bae T.; Schneewind O.; Takeuchi F.; Hiramatsu K. Genome sequence of Staphylococcus aureus strain newman and comparative analysis of staphylococcal genomes: Polymorphism and evolution of two major pathogenicity islands. J. Bacteriol. 2008, 190, 300–310. 10.1128/jb.01000-07. 17951380 PMC2223734

[ref38] Boyle-Vavra S.; Li X.; Alam M. T.; Read T. D.; Sieth J.; Cywes-Bentley C.; Dobbins G.; David M. Z.; Kumar N.; Eells S. J.; et al. USA300 and USA500 clonal lineages of Staphylococcus aureus do not produce a capsular polysaccharide due to conserved mutations in the cap5 locus. mBio 2015, 6, e02585-14 10.1128/mBio.02585-14. 25852165 PMC4453534

[ref39] He J.; Baldini R. L.; Déziel E.; Saucier M.; Zhang Q.; Liberati N. T.; Lee D.; Urbach J.; Goodman H. M.; Rahme L. G. The broad host range pathogen Pseudomonas aeruginosa strain PA14 carries two pathogenicity islands harboring plant and animal virulence genes. Proc. Natl. Acad. Sci. U.S.A. 2004, 101, 2530–2535. 10.1073/pnas.0304622101. 14983043 PMC356984

[ref40] Salunkhe P.; Smart C. H. M.; Morgan J. A. W.; Panagea S.; Walshaw M. J.; Hart C. A.; Geffers R.; Tümmler B.; Winstanley C. A Cystic Fibrosis Epidemic Strain of Pseudomonas aeruginosa Displays Enhanced Virulence and Antimicrobial Resistance. J. Bacteriol. 2005, 187, 4908–4920. 10.1128/jb.187.14.4908-4920.2005. 15995206 PMC1169510

[ref41] Lam J. S.; Taylor V. L.; Islam S. T.; Hao Y.; Kocíncová D. Genetic and Functional Diversity of Pseudomonas aeruginosa Lipopolysaccharide. Front. Microbiol. 2011, 2, 118 10.3389/fmicb.2011.00118. 21687428 PMC3108286

[ref42] Khadka N. K.; Aryal C. M.; Pan J. Lipopolysaccharide-Dependent Membrane Permeation and Lipid Clustering Caused by Cyclic Lipopeptide Colistin. ACS Omega 2018, 3, 17828–17834. 10.1021/acsomega.8b02260. 30613815 PMC6312645

[ref43] Locock K. E. S.; Michl T. D.; Valentin J. D. P.; Vasilev K.; Hayball J. D.; Qu Y.; Traven A.; Griesser H. J.; Meagher L.; Haeussler M. Guanylated polymethacrylates: A class of potent antimicrobial polymers with low hemolytic activity. Biomacromolecules 2013, 14, 4021–4031. 10.1021/bm401128r. 24099527

[ref44] Gonzalez M. R.; Ducret V.; Leoni S.; Fleuchot B.; Jafari P.; Raffoul W.; Applegate L. A.; Que Y. A.; Perron K. Transcriptome analysis of Pseudomonas aeruginosa cultured in human burn wound exudates. Front. Cell. Infect. Microbiol. 2018, 8, 39 10.3389/fcimb.2018.00039. 29535973 PMC5835353

[ref45] Schmidt S.; Röck K.; Sahre M.; Burkhardt O.; Brunner M.; Lobmeyer M. T.; Derendorf H. Effect of protein binding on the pharmacological activity of highly bound antibiotics. Antimicrob. Agents Chemother. 2008, 52, 3994–4000. 10.1128/aac.00427-08. 18779351 PMC2573140

[ref46] Dalhoff A. Seventy-five years of research on protein binding. Antimicrob. Agents Chemother. 2018, 62, e01663-17 10.1128/aac.01663-17. PMC5786787 29158276

[ref47] Thoma L. M.; Boles B. R.; Kuroda K. Cationic methacrylate polymers as topical antimicrobial agents against staphylococcus aureus nasal colonization. Biomacromolecules 2014, 15, 2933–2943. 10.1021/bm500557d. 25010735 PMC4130249

[ref48] Snoussi M.; Talledo J. P.; Del Rosario N. A.; Ha B. Y.; Košmrlj A.; Taheri-Araghi S. Heterogeneous Absorption of Antimicrobial Peptide LL37 in Escherichia coli Cells Enhances Population Survivability. eLife 2018, 7, e38174 10.7554/elife.38174. 30560784 PMC6298785

[ref49] Simpson D. H.; Hapeshi A.; Rogers N. J.; Brabec V.; Clarkson G. J.; Fox D. J.; Hrabina O.; Kay G. L.; King A. K.; Malina J.; et al. Metallohelices that kill Gram-negative pathogens using intracellular antimicrobial peptide pathways. Chem. Sci. 2019, 10, 9708–9720. 10.1039/c9sc03532j. 32015803 PMC6977464

[ref50] Lam J. S.; Taylor V. L.; Islam S. T.; Hao Y.; Kocíncová D. Genetic and functional diversity of Pseudomonas aeruginosa lipopolysaccharide. Front. Microbiol. 2011, 2, 118 10.3389/fmicb.2011.00118. 21687428 PMC3108286

[ref51] Paredes-Gamero E. J.; Martins M. N. C.; Cappabianco F. A. M.; Ide J. S.; Miranda A. Characterization of dual effects induced by antimicrobial peptides: Regulated cell death or membrane disruption. Biochim. Biophys. Acta, Gen. Subj. 2012, 1820, 1062–1072. 10.1016/j.bbagen.2012.02.015. 22425533

[ref52] Barlow P. G.; Beaumont P. E.; Cosseau C.; Mackellar A.; Wilkinson T. S.; Hancock R. E. W.; Haslett C.; Govan J. R. W.; Simpson A. J.; Davidson D. J. The human cathelicidin LL-37 preferentially promotes apoptosis of infected airway epithelium. Am. J. Respir. Cell Mol. Biol. 2010, 43, 692–702. 10.1165/rcmb.2009-0250oc. 20097832 PMC2993089

[ref53] Kuroda K.; Caputo G. A.; DeGrado W. F. The role of hydrophobicity in the antimicrobial and hemolytic activities of polymethacrylate derivatives. Chemistry 2009, 15, 1123–1133. 10.1002/chem.200801523. 19072946 PMC3814040

[ref54] Wang Y.; Xu J.; Zhang Y.; Yan H.; Liu K. Antimicrobial and Hemolytic Activities of Copolymers with Cationic and Hydrophobic Groups: A Comparison of Block and Random Copolymers. Macromol. Biosci. 2011, 11, 1499–1504. 10.1002/mabi.201100196. 21818858

[ref55] Allolio C.; Magarkar A.; Jurkiewicz P.; Baxová K.; Javanainen M.; Mason P. E.; Šachl R.; Cebecauer M.; Hof M.; Horinek D.; et al. Arginine-rich cell-penetrating peptides induce membrane multilamellarity and subsequently enter via formation of a fusion pore. Proc. Natl. Acad. Sci. U.S.A. 2018, 115, 11923–11928. 10.1073/pnas.1811520115. 30397112 PMC6255155

[ref56] Berger J. Preclinical testing on insects predicts human haematotoxic potentials. Lab. Anim. 2009, 43, 328–332. 10.1258/la.2008.007162. 19505933

[ref57] Ignasiak K.; Maxwell A. Galleria mellonella (greater wax moth) larvae as a model for antibiotic susceptibility testing and acute toxicity trials. BMC Res. Notes 2017, 10, 428 10.1186/s13104-017-2757-8. 28851426 PMC5576310

[ref58] Desbois A. P.; Coote P. J. Utility of Greater Wax Moth Larva (Galleria mellonella) for Evaluating the Toxicity and Efficacy of New Antimicrobial Agents. Adv. Appl. Microbiol. 2012, 78, 25–53. 10.1016/B978-0-12-394805-2.00002-6. 22305092

[ref59] Moya-Andérico L.; Vukomanovic M.; Cendra M. d. M.; Segura-Feliu M.; Gil V.; del Río J. A.; Torrents E. Utility of Galleria mellonella larvae for evaluating nanoparticle toxicology. Chemosphere 2021, 266, 129235 10.1016/j.chemosphere.2020.129235. 33316472

[ref60] Allegra E.; Titball R. W.; Carter J.; Champion O. L. Galleria mellonella larvae allow the discrimination of toxic and non-toxic chemicals. Chemosphere 2018, 198, 469–472. 10.1016/j.chemosphere.2018.01.175. 29425947

[ref61] Zhang L.; Dhillon P.; Yan H.; Farmer S.; Hancock R. E. W. Interactions of bacterial cationic peptide antibiotics with outer and cytoplasmic membranes of Pseudomonas aeruginosa. Antimicrob. Agents Chemother. 2000, 44, 3317–3321. 10.1128/aac.44.12.3317-3321.2000. 11083634 PMC90199

[ref62] Xiong F.; Dai X.; Li Y. X.; Wei R.; An L.; Wang Y.; Chen Z. Effects of the antimicrobial peptide L12 against multidrug-resistant Staphylococcus aureus. Mol. Med. Rep. 2019, 19, 3337–3344. 10.3892/mmr.2019.9988. 30816474

[ref63] Klubthawee N.; Adisakwattana P.; Hanpithakpong W.; Somsri S.; Aunpad R. A novel, rationally designed, hybrid antimicrobial peptide, inspired by cathelicidin and aurein, exhibits membrane-active mechanisms against Pseudomonas aeruginosa. Sci. Rep. 2020, 10, 9117 10.1038/s41598-020-65688-5. 32499514 PMC7272617

[ref64] Hobson L. J.; Feast W. J. Poly(amidoamine) hyperbranched systems: Synthesis, structure and characterization. Polymer 1999, 40, 1279–1297. 10.1016/s0032-3861(98)00268-7.

[ref65] Martin L.; Peltier R.; Kuroki A.; Town J. S.; Perrier S. Investigating Cell Uptake of Guanidinium-Rich RAFT Polymers: Impact of Comonomer and Monomer Distribution. Biomacromolecules 2018, 19, 3190–3200. 10.1021/acs.biomac.8b00146. 29890077

[ref66] Keddie D. J.; Moad G.; Rizzardo E.; Thang S. H. RAFT agent design and synthesis. Macromolecules 2012, 45, 5321–5342. 10.1021/ma300410v.

[ref67] Patel J. B.; Bradford A. P.; Eliopoulos M. G.; Hindler A. J.; Jenkins G. S.; Lewis S. J.; Limbago B.; Miller A. L.; Nicolau P. D.; Pwell M.; Swenson M. J.; Traczewski M. M.; Turnidge J. D.; Weinstein P. M. L. B. Methods for Dilution Antimicrobial Susceptibility Tests for Bacteria that Grow Aerobically; CLSI (Clinical and Laboratory Standards Institute), 2015. Approved Standard—Tenth Edition; CLSI Document M07-A10.

[ref68] Sovadinova I.; Palermo E. F.; Huang R.; Thoma L. M.; Kuroda K. Mechanism of polymer-induced hemolysis: Nanosized pore formation and osmotic lysis. Biomacromolecules 2011, 12, 260–268. 10.1021/bm1011739. 21166383

[ref69] Banerjee N.; Sengupta S.; Roy A.; Ghosh P.; Das K.; Das S. Functional alteration of a dimeric insecticidal lectin to a monomeric antifungal protein correlated to its oligomeric status. PLoS One 2011, 6, e18593 10.1371/journal.pone.0018593. 21490929 PMC3072408

[ref70] Maguire R.; Duggan O.; Kavanagh K. Evaluation of Galleria mellonella larvae as an in vivo model for assessing the relative toxicity of food preservative agents. Cell Biol. Toxicol. 2016, 32, 209–216. 10.1007/s10565-016-9329-x. 27122324

