# Supplementary material for: Evaluation of the Antimicrobial Activity in Host-Mimicking Media and In Vivo Toxicity of Antimicrobial Polymers as Functional Mimics of AMPs
Source: ACS Appl Mater Interfaces. 2022 Jul 12;14(29):32855–68. doi: 10.1021/acsami.2c05979 (PMC9335526; doi:10.1021/acsami.2c05979)
Supplement: Supplementary file 1 — am2c05979_si_001.pdf [file am2c05979_si_001.pdf]

Supporting information

Evaluation of the antimicrobial activity in host-mimicking media and *in vivo* toxicity of antimicrobial polymers as functional mimics of AMPs

Ramón Garcia Maset[a], *Alexia Hapeshi*[b], *Stephen C. L. Hall*[b,c], *Robert M. Dalgliesh*[c], *Freya Harrison**[d], *Sébastien Perrier**[a,b,e]

[a] Warwick Medical School, University of Warwick, Coventry, CV4 7AL, UK

[b] Department of Chemistry, University of Warwick, Coventry, CV4 7AL, UK

[c] ISIS Neutron and Muon Source, Rutherford Appleton Laboratory, Didcot, OX11 0DE, UK

[d] School of Life Sciences, University of Warwick, Coventry, CV4 7AL, UK

[e] Faculty of Pharmacy and Pharmaceutical Sciences, Monash University, Parkville, Victoria 3052, Australia

* F.Harrison@warwick.ac.uk; s.perrier@warwick.ac.uk

Synthesis and characterization

Nuclear magnetic resonance (NMR) spectroscopy

^{1}H NMR spectra were recorded on Bruker Avance 300 and 400 spectrometers (300 MHz, 400 MHz respectively) at 300 K. Data analysis was performed using Mestrenova.

Synthesis of diBoc-GEAM

diBoc-GEAM was synthesised according to the literature in two steps summarized below.[1]

Step 1: Synthesis of 2-[1,3-Bis(tert-butoxycarbonyl)guanidine]ethylamine

1,3-Bis(*tert*-butoxycarbonyl)-2-methyl-2-thiopseudourea (6.5 g, 27.6 mmol) was dissolved in DCM (50 mL) and added dropwise to a solution of ethylenediamine (3.77 g, 4.20 mL, 77.3 mmol) in DCM (60 mL). The reaction mixture was then stirred at RT for 2 hours. Subsequently, the product was washed with 3 x 50 mL of water and 2 x 50 mL of brine and dried over $MgSO_4$. The solvent was removed by rotary evaporation to obtain a white solid (6.76 g, 22.3 mmol, 80 %).

Step 2: Synthesis of 2-[1,3-Bis(tert-butoxycarbonyl)guanidine]ethyl acrylamide (diboc-GEAM)

2-[1,3-Bis(tert-butoxycarbonyl)guanidine]ethylamine (6.56 g, 21.6 mmol) and TEA (2.63 g, 3.62 mL, 26.0 mmol) were dissolved in 150 mL of DCM and cooled down in an ice bath. A solution of acryolyl chloride (1.96 g, 1.7 mL, 21.6 mmol) in DCM (50 mL) was added dropwise to the reaction mixture, which was then stirred at RT overnight. Afterwards, 300 mL of saturated $NaHCO_3$ were added to the reaction mixture and then extracted with 3 x 300 mL of DCM. The organic fractions were collected and dried over $MgSO_4$. The solvent was evaporated and the product was purified by column chromatography (hexane/EtOAc) to yield a white solid (6.4 g, 18.0 mmol, 83 %); mp = 142 °C. ^{1}H NMR ($CDCl_3$, δ): 11.30 (s, 1 H, amide proton), 8.63 (s, 1 H,

NH), 8.06 (s, 1 H, amide proton), 6.23 – 6.04 (m, 2H, vinyl protons), 5.55 – 5.47 (dd, 1 H, vinyl proton), 3.58 (m, 2 H, CH_2), 3.47 (m, 2 H, CH_2), 1.45-1.41 (d, 18 H, CH_3 Boc group protons), 1) as shown in **Figure S1**.

Synthesis of Boc-AEAM

Boc-AEAM was synthesised according to the literature. In two steps as summarised below.[2]

Step 1: Synthesis of N-t-butoxycarbonyl-1,2-diaminoethane

Ethylenediamine (4.41 g, 4.9 mL, 73 mmol) was dissolved in 40 mL of DCM and then transferred into a 2necked 100 mL flask equipped with a condenser, a pressure equalising dropping funnel and a nitrogen inlet. After cooling down the reaction mixture with an icebath, a solution of Boc-anhydride (3.98 g, 18 mmol) in DCM (20 mL) was added dropwise over 2 hours with stirring. Subsequently, the reaction mixture was allowed to warm up to RT and stirred overnight. The solvent was then removed by rotary evaporation and a precipitate, identified as *N,N'*-(bis-*t*-butoxycarbonyl)-1,2-diaminoethane, was observed upon addition of water (50 mL). After filtration, the resulting product was saturated with NaCl and extracted with EtOAc (3 x 60 mL). The combined organic phases were concentrated under reduced pressure resulting in a pale oil. Residual NaCl was removed by dissolution of the oil in $CHCl_3$ and filtration of the solution. The solvent was removed under reduced pressure to give a colourless oil identified as *N-t*-butoxycarbonyl-1,2-diaminoethane (1.51 g, 9 mmol, 50 %).

Step 2: Synthesis of N-t-butoxycarbonyl-N'-acryloyl-1,2-diaminoethane

A solution of acryloyl chloride (0.67 g, 0.6 mL, 7.4 mmol) in $CHCl_3$ (30 mL) was cooled down in an ice bath. Subsequently, a solution of NEt_3 (0.63 g, 0.9 mL, 6.2 mmol) and *N-t*-butoxycarbonyl-1,2-diaminoethane (1 g, 6.2 mmol) in $CHCl_3$ (15 mL) was added dropwise to

the reaction mixture over a period of an hour and a half. The reaction mixture was then allowed to warm up to RT and stirred for an hour before the solvent was removed under reduced pressure. The residue was washed with water (20 mL) and extracted with $CHCl_3$ (3 x 20 mL). The organic fractions were collected, combined and the solvent was removed under vacuum to obtain *N*-*t*-butoxycarbonyl-*N'*-acryloyl-1,2-diaminoethane as a white powder. The product was recrystallized in Et_2O yielding white crystals (1.04 g, 4.9 mmol, 80 %). ^{1}H NMR ($CDCl_3$): $\delta = 6.43$ (bs, 1H, amide proton), 6.24-6.615 (m, 2H, vinyl protons) 5.65 (d, 1H, vinyl proton), 4.93 (bs, 1H, amide proton), 3.35(m, 2H, CH2), 3,31 (m, 2H, CH2), 1.36 (s, 9H, CH3 boc group protons)) as shown in **Figure S2.**

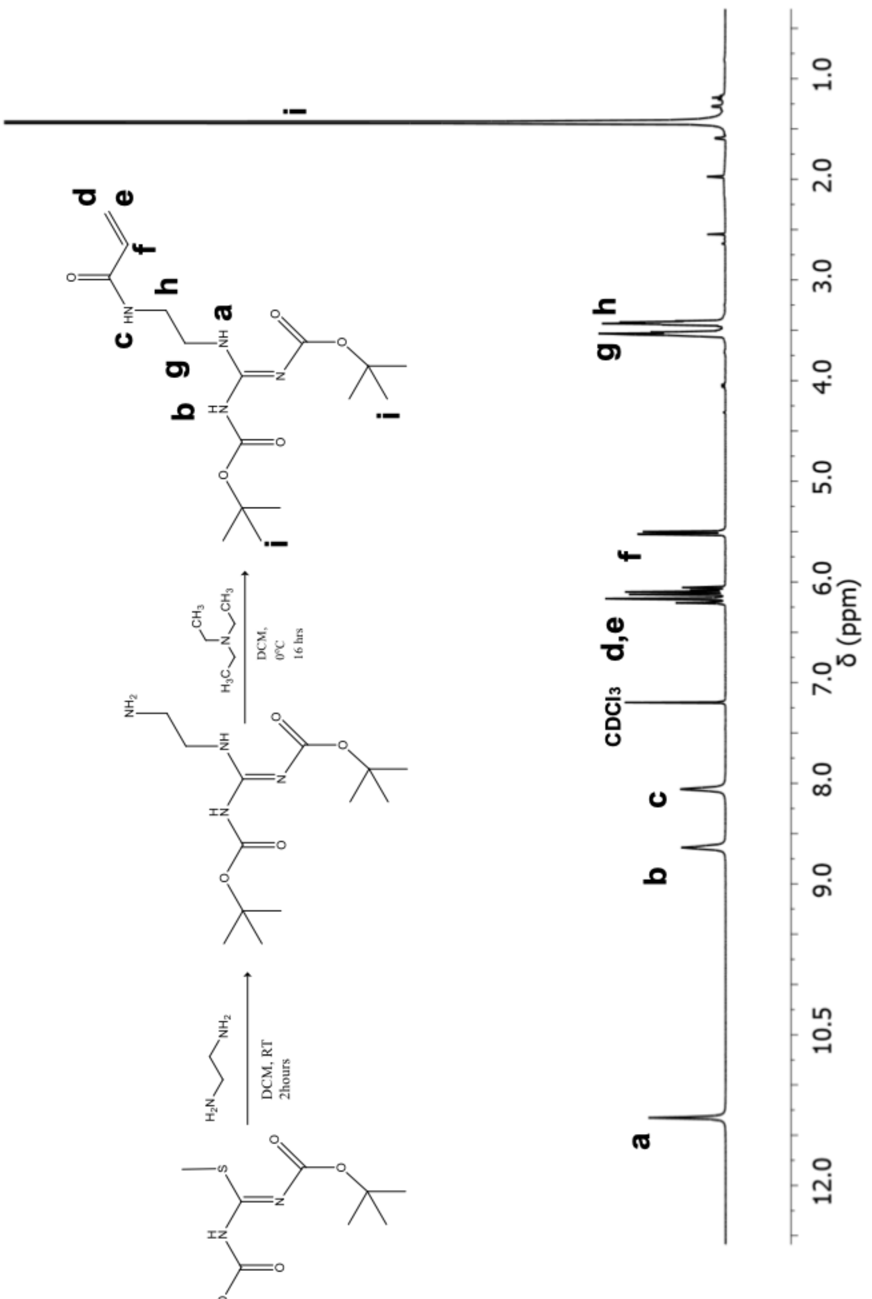

Figure S1. ^{1}H NMR spectrum of diBoc-GEAM monomer in d-chloroform.

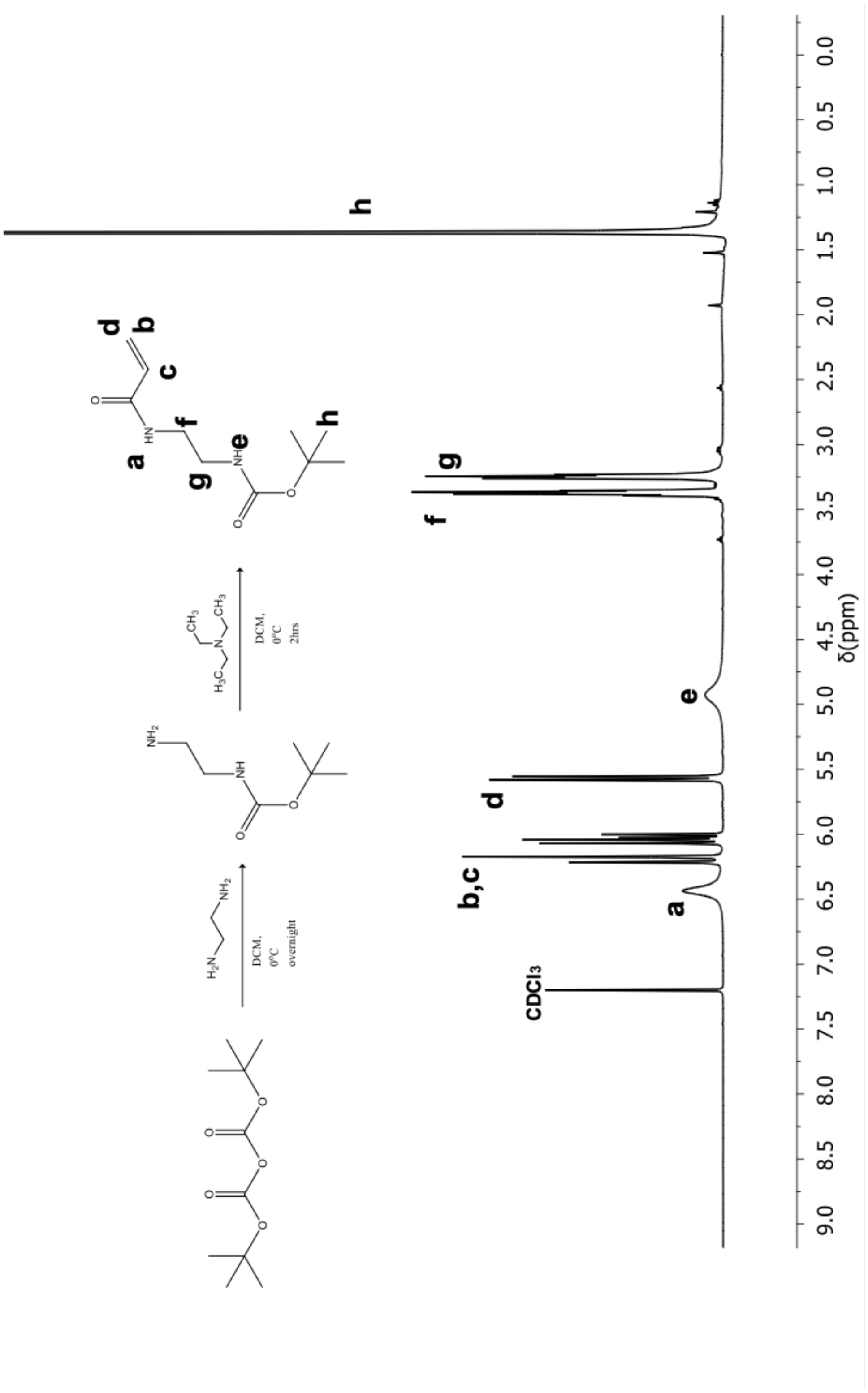

Figure S2. ¹H NMR spectrum of Boc-AEAM monomer in $CDCl_3$.

Diblock and triblock copolymer synthesis by RAFT polymerisation

The following general procedure was used for all RAFT polymerisations.

Synthesis of the first block

Monomer, initiator (VA-044), CTA (PABTC) and solvents (80% dioxane, 20 % water) were introduced in a vial with a magnetic stirrer and a rubber septum. The solution was degassed with nitrogen approximately for 20 min. Then, the reaction vial was placed in an oil bath at 46 °C for 6 hours to perform the RAFT polymerization. After 6h, the test tube was withdrawn from the oil bath and a sample was taken for 1H NMR (Figure S3) and GPC analysis. (Figure S9).

Synthesis of subsequent blocks

The reaction vial with the reaction mixture was opened and additional monomer, initiator and solvent were introduced. The reaction vial was sealed with a rubber septum and degassed with nitrogen approximately for 20 min. Then, the reaction vial was placed in an oil bath at 46 °C for 6 hours to perform the RAFT polymerization. After 6 h, the test tube was withdrawn from the oil bath and a sample was taken for 1H NMR (Figure S4-8) and GPC analysis (Figure S9, Table S2). The quantity of reagents needed for the diblock and triblock copolymers is summarized in Table S1.

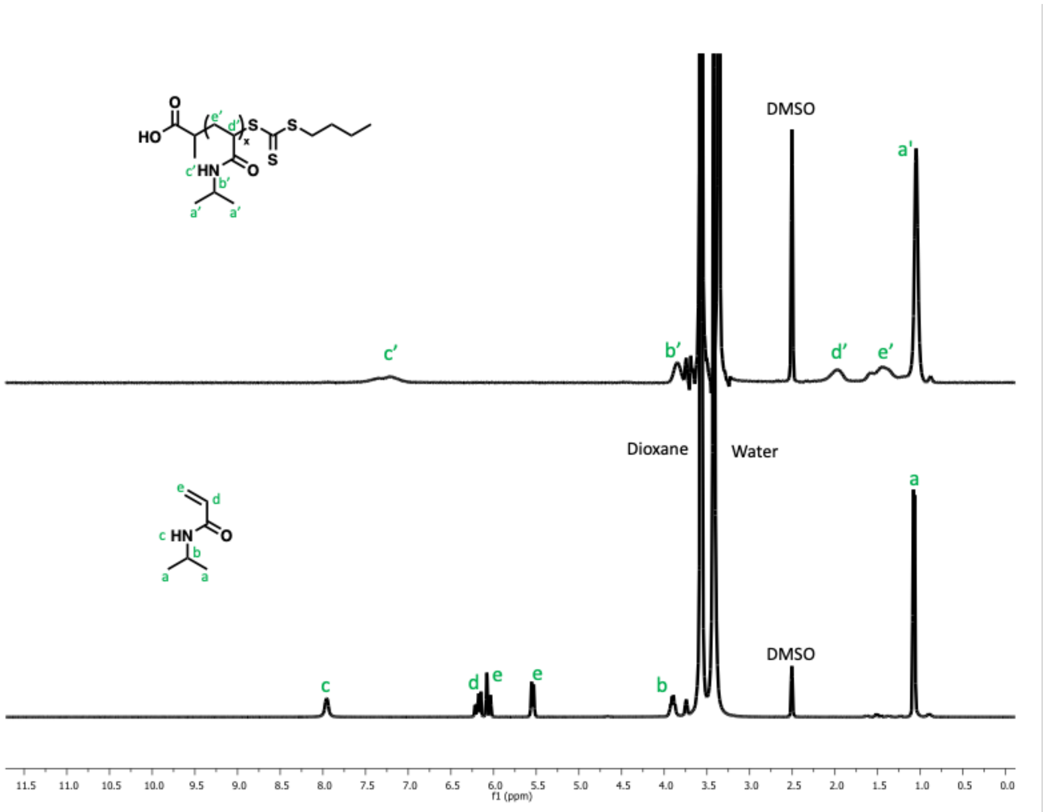

Figure S3. ^1H NMR spectra in DMSO-d_6 of the first chain extension (NIPAM monomer) of g-D50.

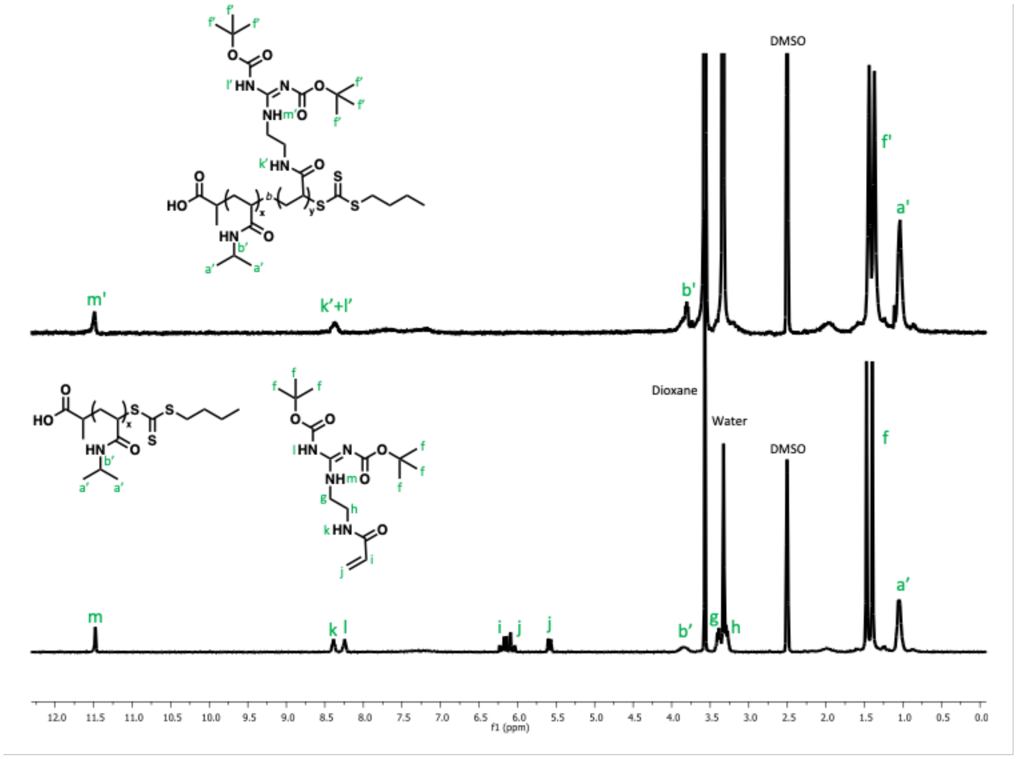

Figure S4. ^{1}H NMR spectra in DMSO-d_6 of the second chain extension (Boc-GEAM) of g-D50.

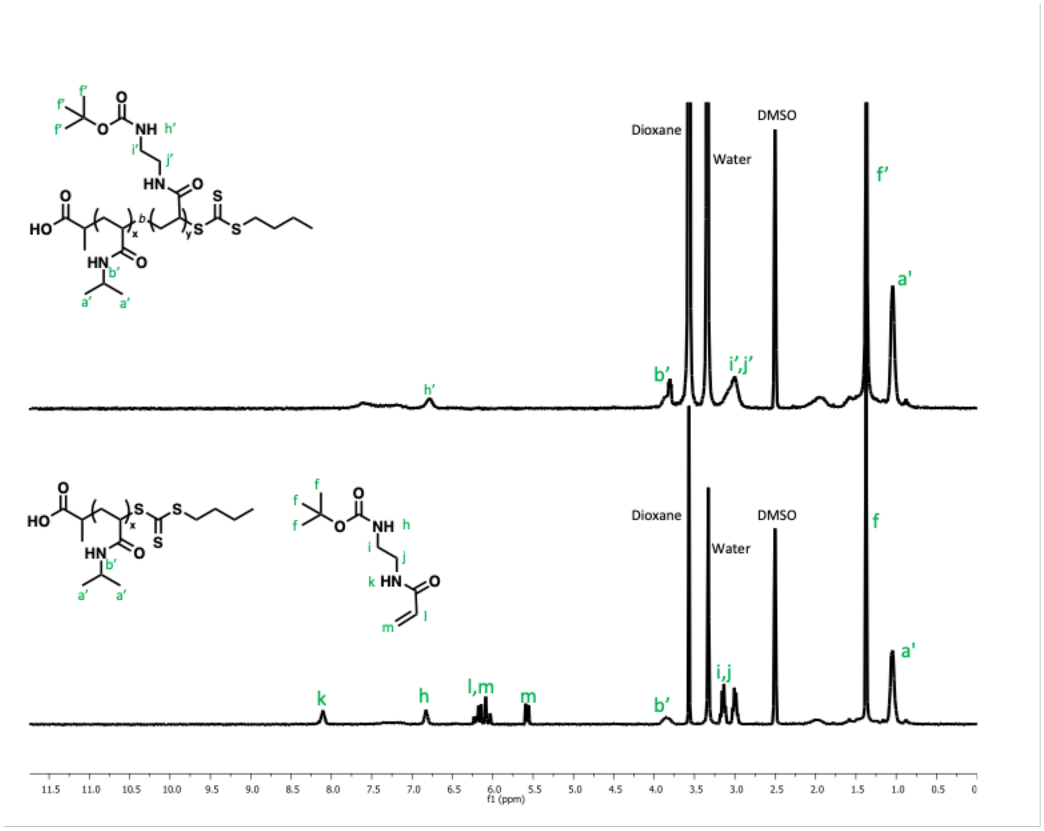

Figure S5. ^{1}H NMR spectra in DMSO-d_6 of the second chain extension (Boc-AEAM) of a-D50.

Table S1. Reactions conditions for the RAFT polymerization of the cationic copolymers.

Polymer	g-D50	g-T100(1)	g-T100(2)	a-D50	a-T100(1)	a-T100(2)
Monomer 1	NIPAM	NIPAM	diBoc-GEAM	Boc AEM	NIPAM	Boc AEM
Monomer 2	diBoc-GEAM	diBoc-GEAM	NIPAM	NIPAM	Boc AEM	NIPAM
Monomer 3	-	NIPAM	diBoc-GEAM	-	NIPAM	Boc AEM
$DP_{targeted\ Monomer1}$	35	35	15	35	35	15
$DP_{targeted\ Monomer2}$	15	30	70	15	30	70
$DP_{targeted\ Monomer3}$	-	35	15	-	35	15
$m_{monomer1,added}$ (mg)	282.9	79.21	142.5	282.9	113.1	107.1
$m_{monomer2,added}$ (mg)	381.8	213	211	229.5	183.6	264
$m_{monomer3,added}$ (mg)	-	79.21	142.5	-	113.1	107
$m_{CTA,added}$ (mg)	17.02	4.76	6.35	17.02	6.8	7.9
$m_{VA\text{-}044added}$ (mg)	4.18	2.93	2.39	4.18	3.66	3.23
$V_{dioxane,added}$ (µL)	2143	1400	1067	2143	2000	1556
$V_{water,added}$ (µL)	538	350	272	538	500	400
V_{total}[c] (µL)	2681	1750	1339	2681	2500	1956
$[CTA]_0/[VA\text{-}044]_0$	11	7	11	11	7	11

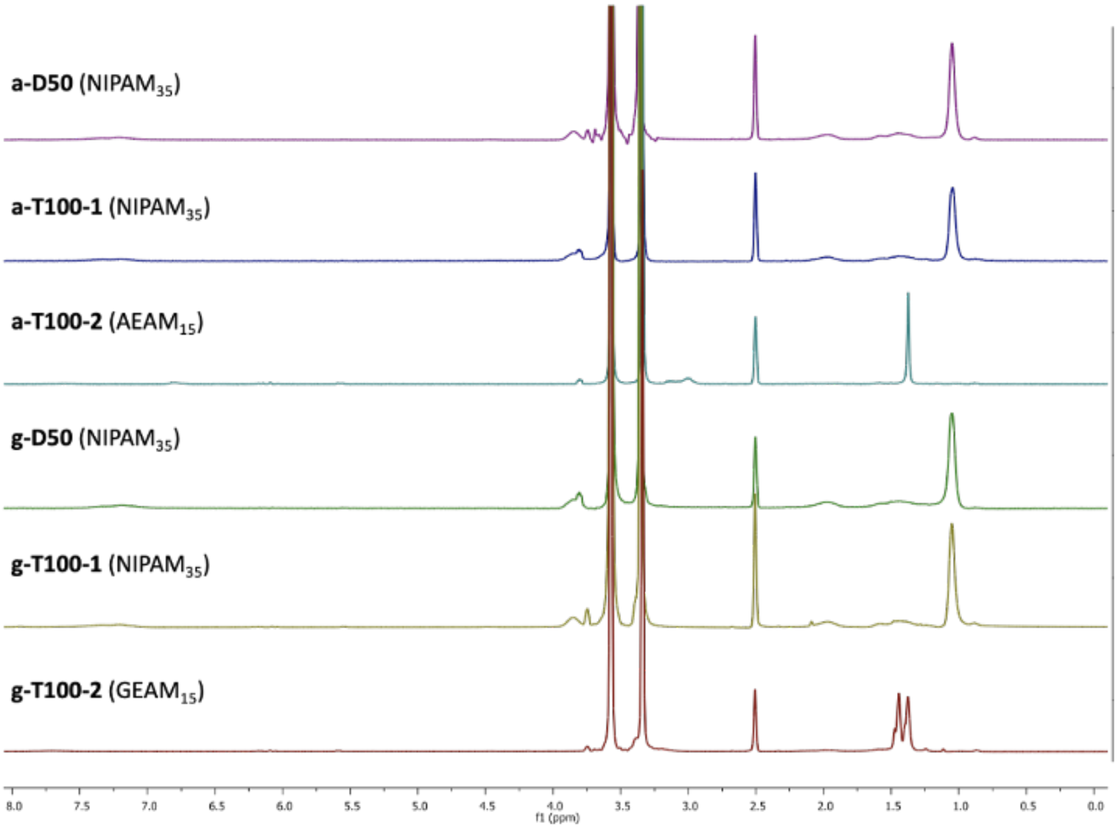

Figure S6. ^{1}H NMR spectra in DMSO-d6 of the first block extensions.

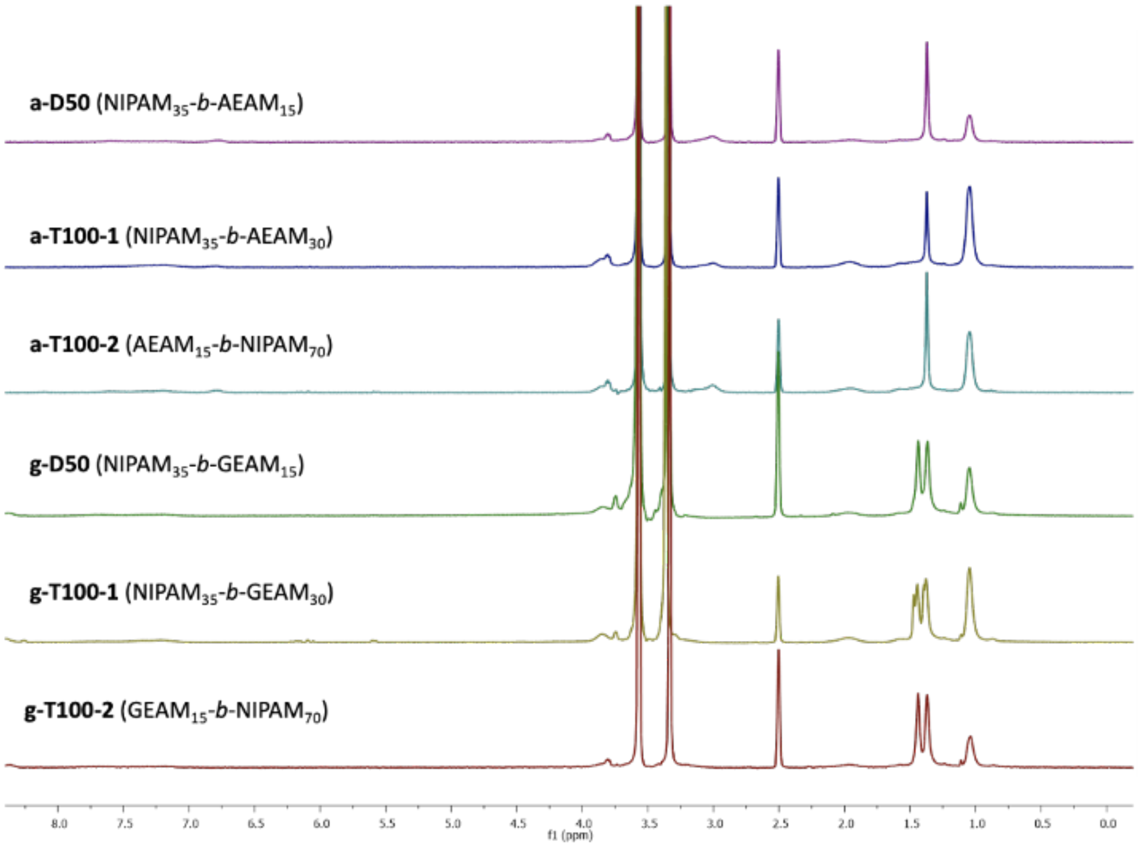

Figure S7. ^{1}H NMR spectra in DMSO-d_6 of the second block extensions

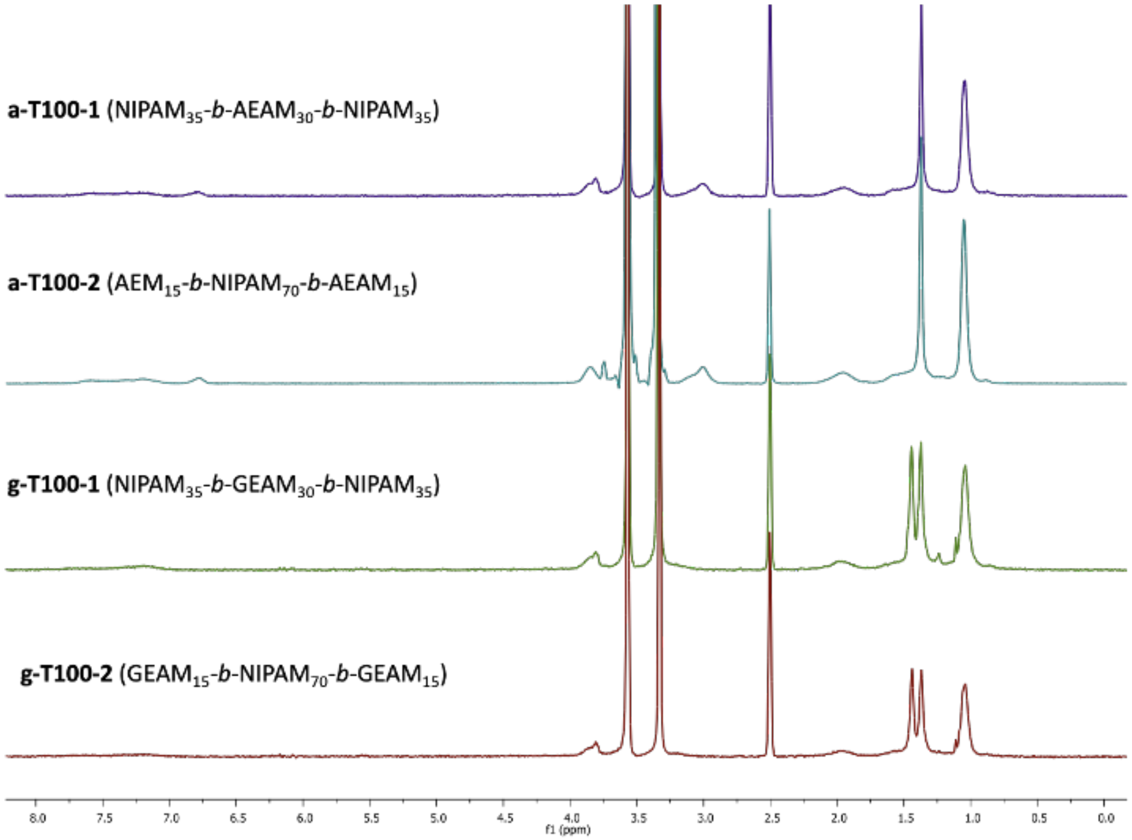

Figure S8. 1H NMR spectra in DMSO-d6 of the third block extensions.

<u>Calculation of $M_{n,th}$</u>

The theoretical number average molar mass ($M_{n,th}$) was calculated as

$$M_{\mathrm{n,th}} = \rho M_{\mathrm{M}} \frac{[\mathrm{M}]_0}{[\mathrm{CTA}]_0} + M_{\mathrm{CTA}}, \tag{1.1}$$

where $[M]_0$ and $[CTA]_0$ are the initial concentrations of the monomer and the chain transfer agent respectively, ϱ is the monomer conversion as determined by ^{1}H NMR, and M_M and M_{CTA} are the molar masses of the monomer and the chain transfer agent respectively.

Size exclusion chromatography (SEC)

2 mg mL^{-1} dilutions of each polymer in DMF containing 0.1 % (w:vt) LiBr were incubated for 16 h at room temperature. Analyte samples were filtered through a nylon membrane with 0.22 μm pore size before injection. An Agilent PL50 instrument equipped with differential refractive index (DRI) and UV detectors was used for all the measurements. The system was equipped with two x PolarGel M columns (300 x 7.5 mm) and a PolarGel 5 μm guard column, connected in series. The eluent was DMF containing 0.1 % LiBr. All experiments were performed at a flow rate of 1ml min^{-1} at 50 °C. Experimental molar mass ($M_{n,SEC}$) and dispersity (*Đ*) values of synthesized polymers were determined by comparison with poly(methyl methacrylate) standards (Agileny EasyVials) using using Agilent GPC/SEC software. GPC traces are shown in **Figure S9** and dispersity (*Đ*) in **Table S2**.

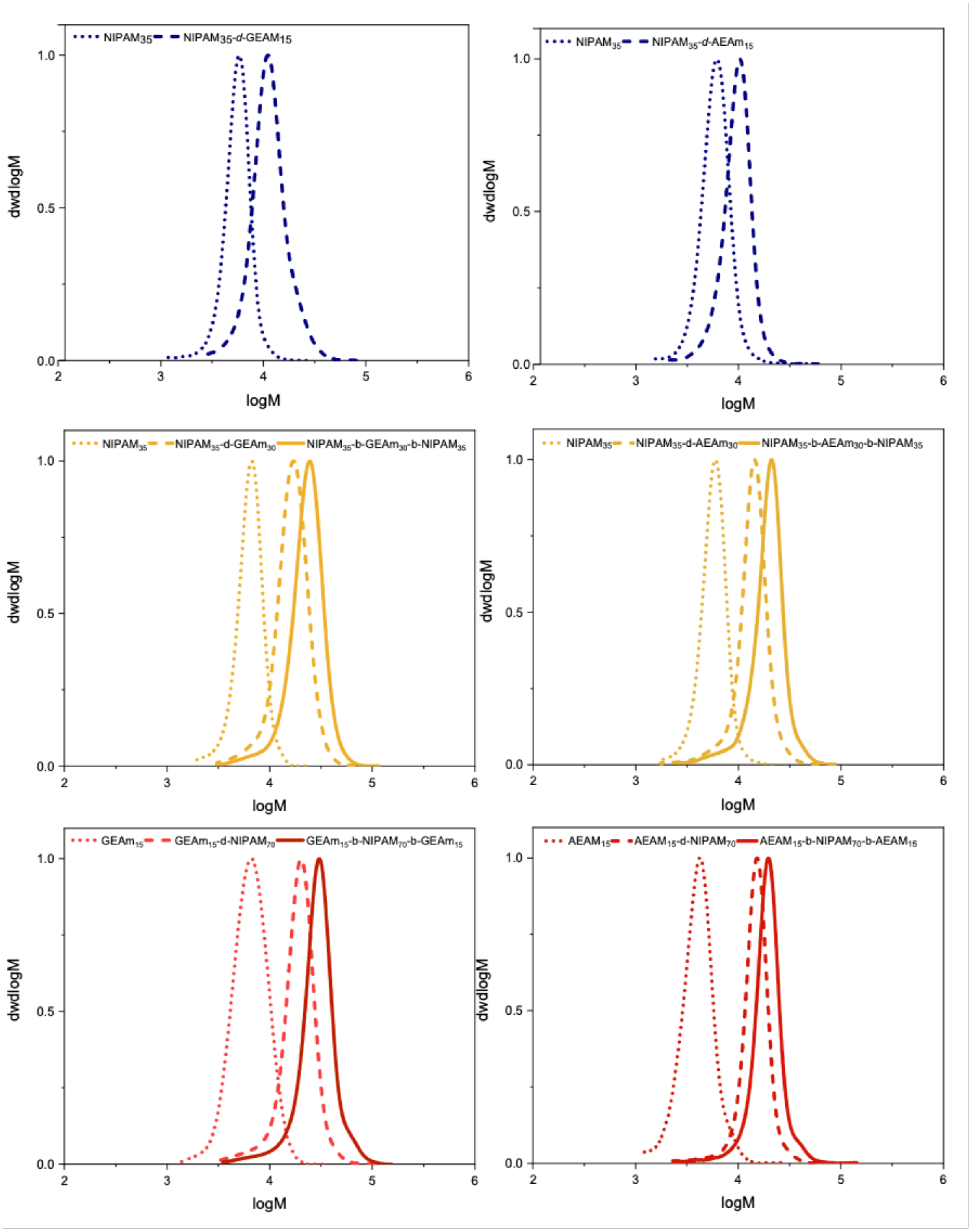

Figure S9. GPC traces in DMF of the Boc-protected copolymers. Dots represent the first block extension, dash the second block extension and lines the third block extension

Table S2. 1H NMR and GPC analyses of the Boc-protected polymers in each chain extensions, and HPLC analysis of the final deprotected copolymers.

	Monomer 1st block	Target DP[a]	$M_{n,theo}$ $M_{n,GPC}$ (g mol^{-1})	Đ	Monomer 2nd block	Target DP[a]	$M_{n,theo}$ $M_{n,GPC}$ (g mol^{-1})	Đ	Monomer 3rd block	Target DP[a]	$M_{n,theo}$ $M_{n,GPC}$ (g mol^{-1})	Đ	Retention time (min)[b]
**g-D50**	NIPAM	35	4200<br>5200	1.11	diBoc GEAM	15	9800<br>10400	1.19	-	-	-	-	24.58
**g-T100-1**	NIPAM	35	4200<br>6000	1.11	diBoc GEAM	30	15600<br>14900	1.16	NIPAM	35	19900<br>20500	1.18	26.41
**g-T100-2**	diBoc GEAM	15	5600<br>5100	1.16	NIPAM	70	13500<br>16900	1.18	diBoc GEAM	15	19900<br>21000	1.21	27.01
**a-D50**	NIPAM	35	4200<br>5500	1.09	Boc AEAM	15	7400<br>9300	1.11	-	-	-	-	25.56
**a-T100-1**	NIPAM	35	4200<br>5400	1.09	Boc AEAM	30	10699<br>12400	1.16	NIPAM	35	14600<br>17300	1.18	25.94
**a-T100-2**	Boc AEAM	15	3400<br>3700	1.15	NIPAM	70	11400<br>13500	1.13	Boc AEAM	15	14600<br>17400	1.17	28.37

[a] Determined by 1H NMR (> 99% conversion), [b] determined by HPLC

Polymer-dye conjugation

The polymer g-D50 (70 mg, 9.47 µmol, 1 eq.) was dissolved in 0.5 mL of anhydrous DMF. N,N-Diisopropylethylamine (DIPEA) (3.67 mg, 28.42 µmol, 3 eq.) and (1-[Bis(dimethylamino)methylene]-1H-1,2,3-triazolo[4,5-b]pyridinium 3-oxide (HATU) (5.04 mg, 14.21 µmol , 1.5 eq) was added and mixed. Cyanine5 amine (purchased from Lumiprobe GmbH) (9.29 mg, x 14.21 µmol, 1.5 eq.) was added to the polymer solution and stirred overnight in the dark (**Figure S10**).

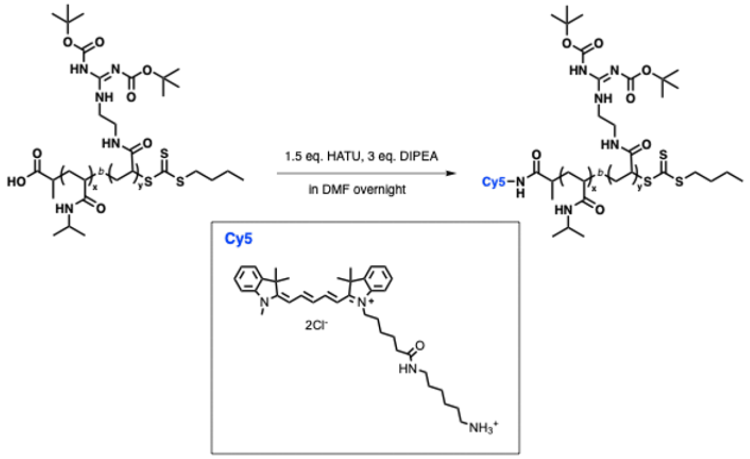

Figure S10. Reaction scheme of polymer-Cy5 conjugation by orthogonal chemistry.

The sample was purified by precipitation in ether and boc groups were deprotected and polymer purification was carried out as described in the next section. Purity was checked by HPLC (**Figure S11).**

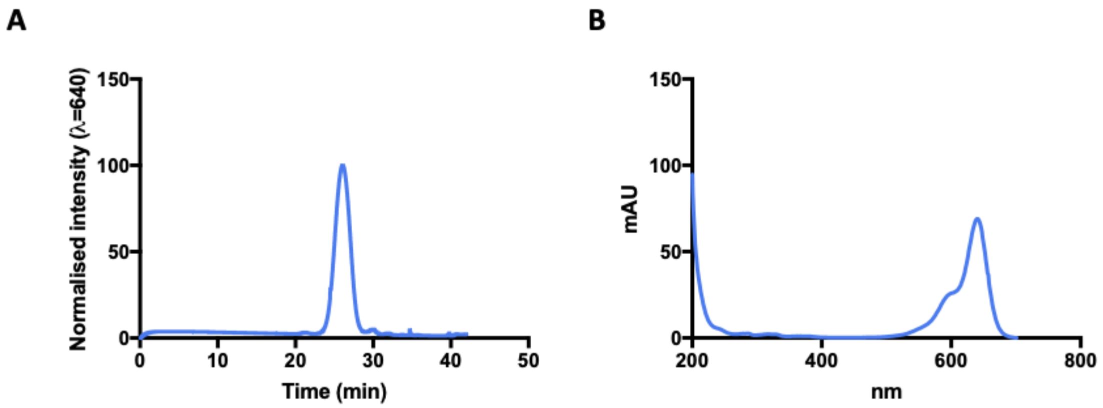

Figure S11. (A) HPLC chromatogram of the gD50-Cy5 with a gradient of 5 to 95 % ACN in 30 minutes using 100 mm C18 column. (B) UV-chromatogram of the eluted peak at x minutes.

Deprotection of the polymers

TFA was added directly to the polymeric solution in DCM, stirred for 3 hours at 40 °C. After the reaction took place, TFA was removed by precipitation in cold diethyl ether three times. In order to replace the TFA counter-ion, the polymers were dialyzed against a NaCl solution, followed by dialysis against distilled water for 3-4 days. Boc-group removal was monitored by 1H NMR and 19F NMR used to monitor for traces of the TFA counterion. (Figures S12-15). Finally, the dialyzed product was freeze-dried and stored at 4 °C.

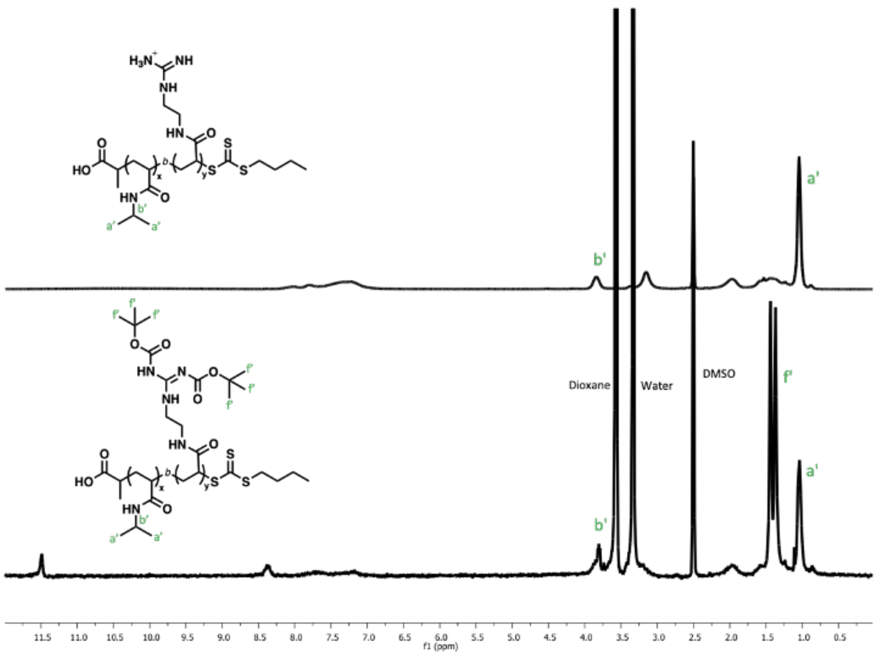

Figure S12. ^1H NMR spectra in DMSO-d_6 of the guanidinium polymer example (g-D50) before (bottom part) and after Boc group removal (upper part).

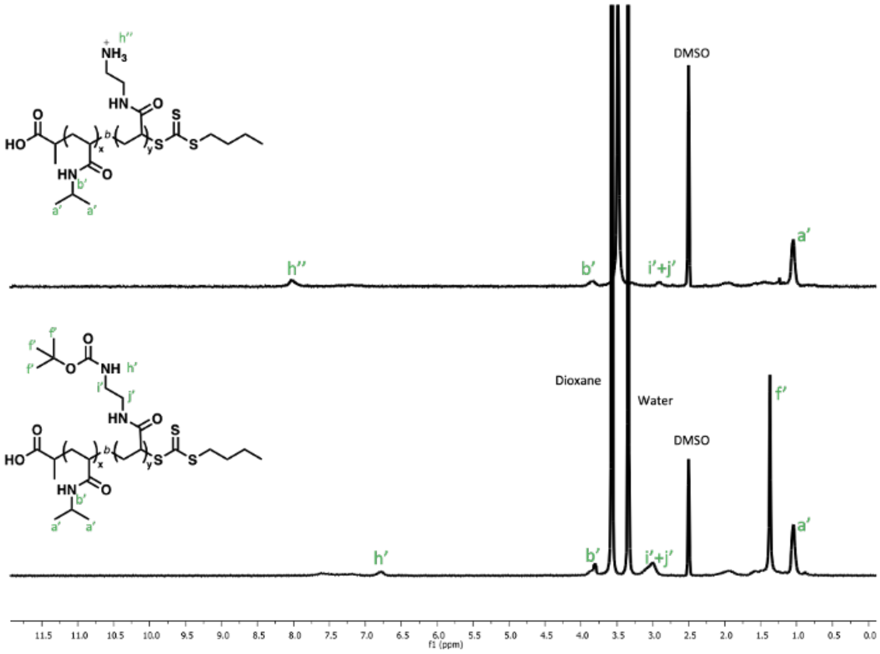

Figure S13. 1H NMR spectra in DMSO-d6 of the ammonium polymer example (a-D50) before (bottom part) and after Boc group removal (upper part).

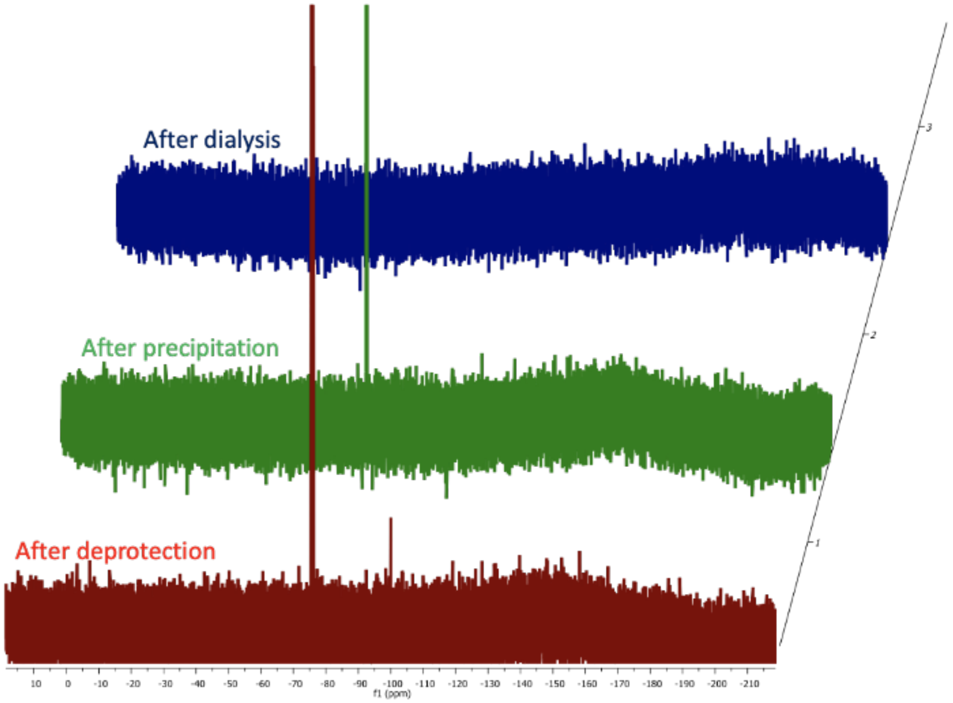

Figure S14. ^{19}F NMR of g-D50 example after deprotection (red), after precipitation (green) and after dialysis (blue).

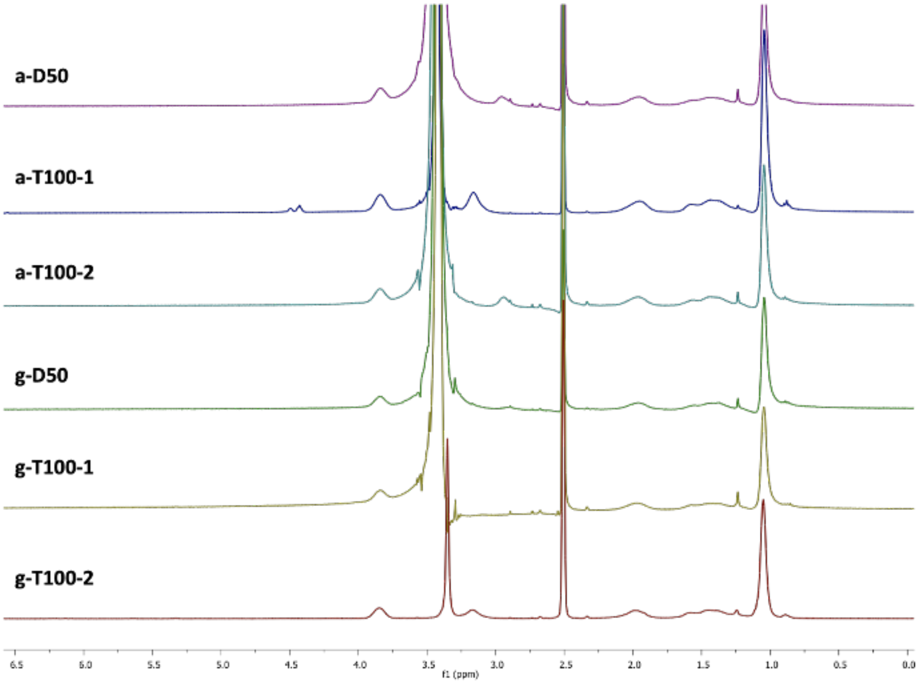

Figure S15. ^{1}H NMR spectra in DMSO-d_6 of the deprotected polymers.

Dynamic light scattering (DLS)

Hydrodynamic diameters (D_h) and size distributions were determined by Dynamic Light Scattering (DLS) using a Zetasizer Nano ZS (Malvern Panalytical) equipped with a 4 mW He-Ne 633 nm laser module. Samples were prepared in PBS to a concentration of 1 mg mL^{-1} before transferring to disposable cuvettes. Samples were equilibrated at 37℃ for 45 seconds prior the measurements. Measurements were performed in a backscattering geometry with a detection angle of 173°. Measurements were repeated three times with an automatic attenuation selection and measurement position. The results were analyzed using Malvern DTS 6.20 software. PDI values were calculated as

$$\mathrm{PDI} = \frac{\sigma^2}{d^2} \qquad (1.2)$$

where σ is the standard deviation, and *d* is the diameter, both obtained from the number-weighted particle size distribution.

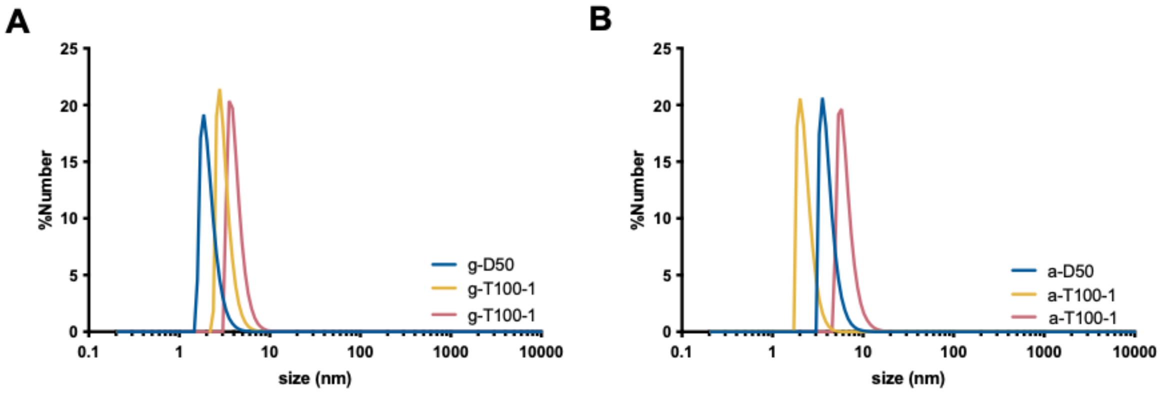

Figure S16. DLS analysis %Number (A) for guanidinium polymers and (B) ammonium counterparts at 37 ^{0}C, in PBS at a polymer concentration of 1 mg mL^{-1}.

Turbidity measurements.

Turbidity analyses for the determination of the transition temperature of each sample were performed using an Agilent Technologies Cary 100 UV-Vis spectrophotometer equipped with an Agilent Technologies Cary temperature controller and an Agilent Technologies 6 x 6 multicell block Peltier. The measurements were performed using Suprasil® quartz cuvettes (Hellman, 100-QS, light path = 10.00 mm) filled with 5 mg mL^{-1} solutions of each polymer in PBS. For each sample, two heating/cooling cycles between 25 and 60 °C were performed with a temperature gradient of 1°C /min at λ = 633 nm. All data were recorded using the Cary WinUV software and elaborated using Prism9.

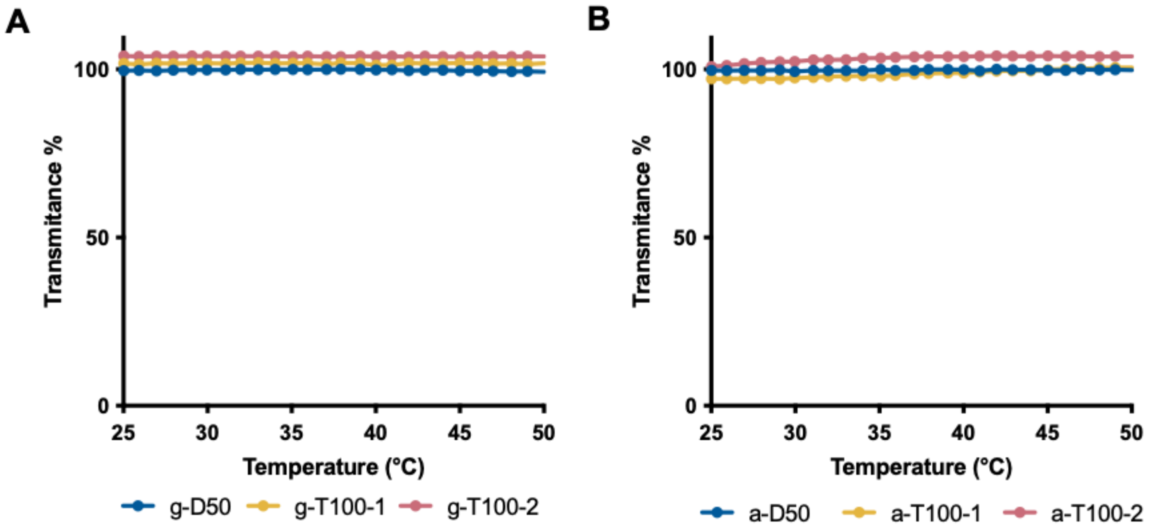

Figure S17. (A) UV-vis transmittance at 633 nm for guanidinium polymers. (B) UV-vis transmittance at 633 nm for ammonium polymers.

High performance liquid chromatography (HPLC)

HPLC was performed using an Agilent 1260 infinity series stack equipped with an Agilent 1260 binary pump and degasser. The flow rate was set to 1.0 mL min^{-1} and samples were injected using Agilent 1260 autosampler with a 20 µL injection volume. The temperature of the column was set

at 37 °C. The HPLC was fitted with a phenomenex Lunar C18 column (150 × 4.6 mm) with 5m packing (100Å). Detection was achieved using an Agilent 1260 variable wavelength detector. UV detection was monitored at λ = 309 nm. Methods were edited and run using Agilent OpenLAB online software and data was analysed using Agilent OpenLAB offline software. Mobile phase solvents used were HPLC grade (ACN was 'far UV') and consisted of mobile phase A: 100 % ACN, 0.04 % TFA; mobile phase B: 100 % water, 0.04 % TFA with a gradient of 5 to 95 % ACN over 30 minutes.

Small-Angle Neutron Scattering (SANS)

SANS experiments were performed using the LARMOR instrument at the ISIS Neutron and Muon Source (STFC Rutherford Appleton Laboratory, Didcot, UK). Each sample was dissolved in PBS, prepared in D_2O, to a concentration of 5 mg mL^{-1} and placed into a 2 mm path length rectangular quartz cuvette. SANS measures the scattered intensity of neutrons from a sample as a function of the momentum transfer, Q, defined as:

$$Q = \frac{4\pi sin\theta}{\lambda} \qquad (1.3)$$

Where 2θ is the scattering angle, and λ is the neutron wavelength. The scattering cross-section was measured using time-of-flight over a Q-range of 0.004 – 0.7 $Å^{-1}$ using a pulsed neutron beam with a wavelength range of 0.9 – 13.3 Å. 2-Dimensional scattering patterns were collected using a ^3He-tube array area detector (600×664 mm, 512×80 pixels) positioned 4.1 m from the sample position with beam dimensions of 6×8mm centred on the detector. All SANS data were reduced using Mantid Workbench software.[3] Briefly, all samples produced isotropic scattering, allowing

radial averaging of the 2-dimensional detector intensities after correcting for detector efficiencies. These data were normalised to the incident flux and the spectrum of the direct beam before subtraction of background spectra (processed identically) collected from PBS in D_2O. These data were then placed on an absolute scale using the scattering from a standard sample (a solid blend of hydrogenous and deuterated polystyrene) in accordance with established procedures.

SANS data were analysed using SasView software (version 5.0.3) using a custom model describing two scattering contributions, a polydisperse Gaussian coil form factor[4] summed with an exponential power law as an approximation to fractal Porod scattering from larger aggregates where the scattering intensity as a function of Q, $I(Q)$ is described by:

$$I(Q) = I_0 \cdot 2\left(\left(1 + (Đ - 1)\frac{Q{R_g}^2}{2Đ - 1}\right)^{\frac{-1}{Đ-1}} + \frac{Q{R_g}^2}{2Đ - 1} - 1\right)\left(\frac{ĐQ{R_g}^2}{2Đ - 1}\right)^{-1} + \frac{C}{Q^d} + Bkg \quad (1.4)$$

Where Đ represents the polydispersity, defined as M_w/M_n, R_g represents the radius of gyration of an individual polymer chain, and I_0 describes the zero-angle intensity, proportional to the volume fraction of non-aggregated polymer chains in solution, χ_{pol}, the molecular volume of a single chain, V_{pol}, and the difference in scattering length density between the polymer chains and bulk solvent, $\Delta\rho$, where

$$I_0 = \chi_{pol} \cdot V_{pol} \cdot \Delta\rho^2 \quad (1.5)$$

The power law component is described by the exponent d, and scaling factor C, proportional to both the radius of gyration of the aggregates (described at inaccessibly low Q) and the proportion

of polymer chains undergoing aggregation. Finally, a constant background is summed into the model to account for the incoherent background.

Table S3. Structural parameters obtained by fitting SANS data of aqueous solutions of cationic block copolymers.

Polymer	I_0 / cm^{-1}	R_g / Å	Đ	Porod Scale	Porod Exponent
**g-D50**	0.071 ± 0.003	29.0 ± 0.9	1.19	3.48×10^{-7} ± 5.69×10^{-7}	2.59 ± 0.34
**g-T100-1**	0.411 ± 0.052	93.6 ± 5.6	1.18	4.33×10^{-8} ± 1.96×10^{-8}	3.07 ± 0.86
**g-T100-2**	0.196 ± 0.014	49.7 ± 1.5	1.21	2.53×10^{-6} ± 4.11×10^{-6}	2.32 ± 0.32
**a-D50**	0.031 ± 0.009	50.8 ± 9.7	1.11	4.22×10^{-4} ± 5.84×10^{-5}	1.49 ± 0.03
**a-T100-1**	0.025 ± 0.005	40.6 ± 5.5	1.17	4.58×10^{-7} ± 1.00×10^{-7}	3.07 ± 0.04
**a-T100-2**	0.22 ± 0.02	63.3 ± 1.9	1.18	4.86×10^{-5} ± 3.03×10^{-5}	1.83 ± 0.12

The Zero-angle intensity, I_0, describes the intensity of the polydisperse Gaussian coil form factor in the absence of any Porod scattering contribution. The polydispersity, Đ, was fixed for each polymer throughout the fitting procedure based on values obtained though SEC analyses.

Biological experiments

Table S4. MIC values against *S. aureus* (USA300 and Newman) in Muller-Hilton media (MHB) and synthetic wound media (SWM). MIC values against *P. aeruginosa* (PA14 and LESB58) in Muller-Hilton media (MHB) and synthetic wound media (SWM) and synthetic cystic fibrosis sputum medium (SCFM). Experiments were carried out in at least 3 independent replicates.

		**USA 300**		**Newman**		**PA14**			**LESB58**		
Cationic charge	Architecture	caMHB	SWM	caMHB	SWM	caMHB	SWM	SCFM	caMHB	SWM	SCFM
Guanidinium	**D50**	128	64	128	32	>512	>512	>512	64	>512	254
	**T100-1**	128	32	128	32	128	>512	16	64	>512	16
	**T100-2**	64	32	128	32	128	>512	16	64	>512	16
Ammonium	**D50**	256	64	256	64	>512	>512	64	32	>512	32
	**T100-1**	256	256	256	128	128	>512	64	128	>512	128
	**T100-2**	256	128	256	64	128	>512	64	32	>512	64

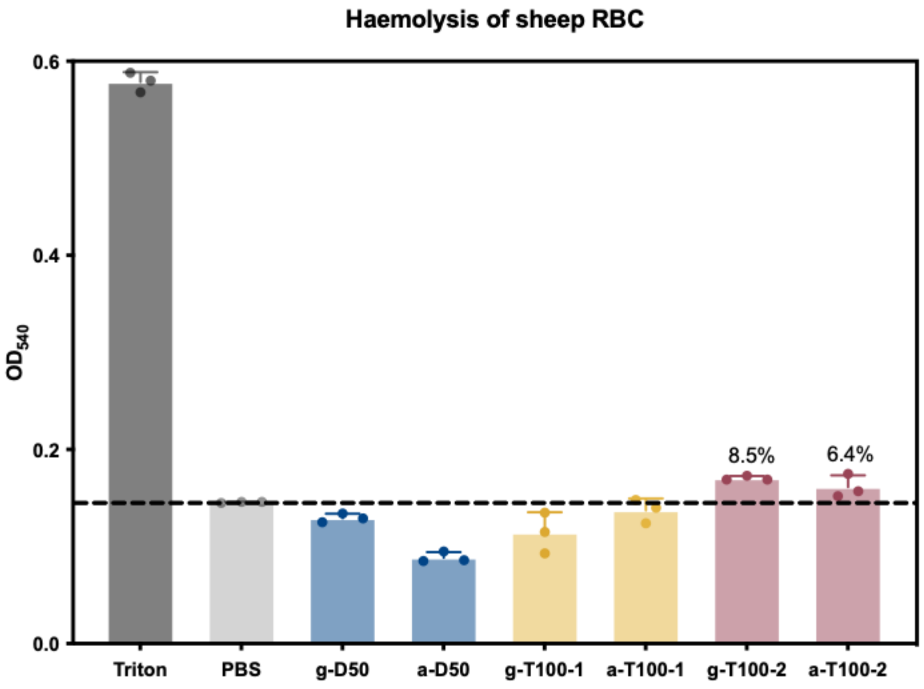

Figure S18. Haemolysis of sheep RBC in the presence of 1 mg mL^{-1} of polymeric materials.

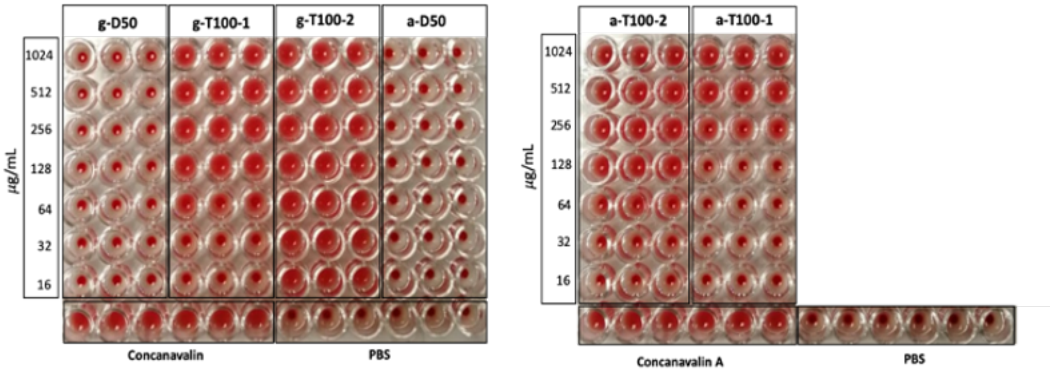

Figure S19. Hemagglutination against RBC. As controls, Convavalin A was used as a positive control, and PBS used as a negative control.

Table S5. a [10]HC values against sheep red blood cells. b Hemagglutination of sheep red blood cells in presence of polymeric solution. Polymers were dissolved in PBS.

		**Haemolysis**	**Hemagglutination**
Cationic charge	Architecture	HC_{10} (μM)	μM
Guanidinium	D50	>138.5	>138.5
	T100-1	>70.6	2.2
	T100-2	>70.6	<1.1
Ammonium	D50	>151.5	>151.5
	T100-1	>77.3	9.7
	T100-2	>77.3	2.4

Table S6. IC50 values against mammal cell lines in vitro (3T3a) and (A549b) measured using an XTT viability assay.

		**3T3**	**A559**
Cationic charge	Architecture	IC_{50}(μM)	IC_{50}(μM)
Guanidinium	D50	34.6	-
	T100-1	2.5	-
	T100-2	1.1	-
Ammonium	D50	>151.5	>151.5
	T100-1	>77.3	>77.3
	T100-2	>77.3	>77.3

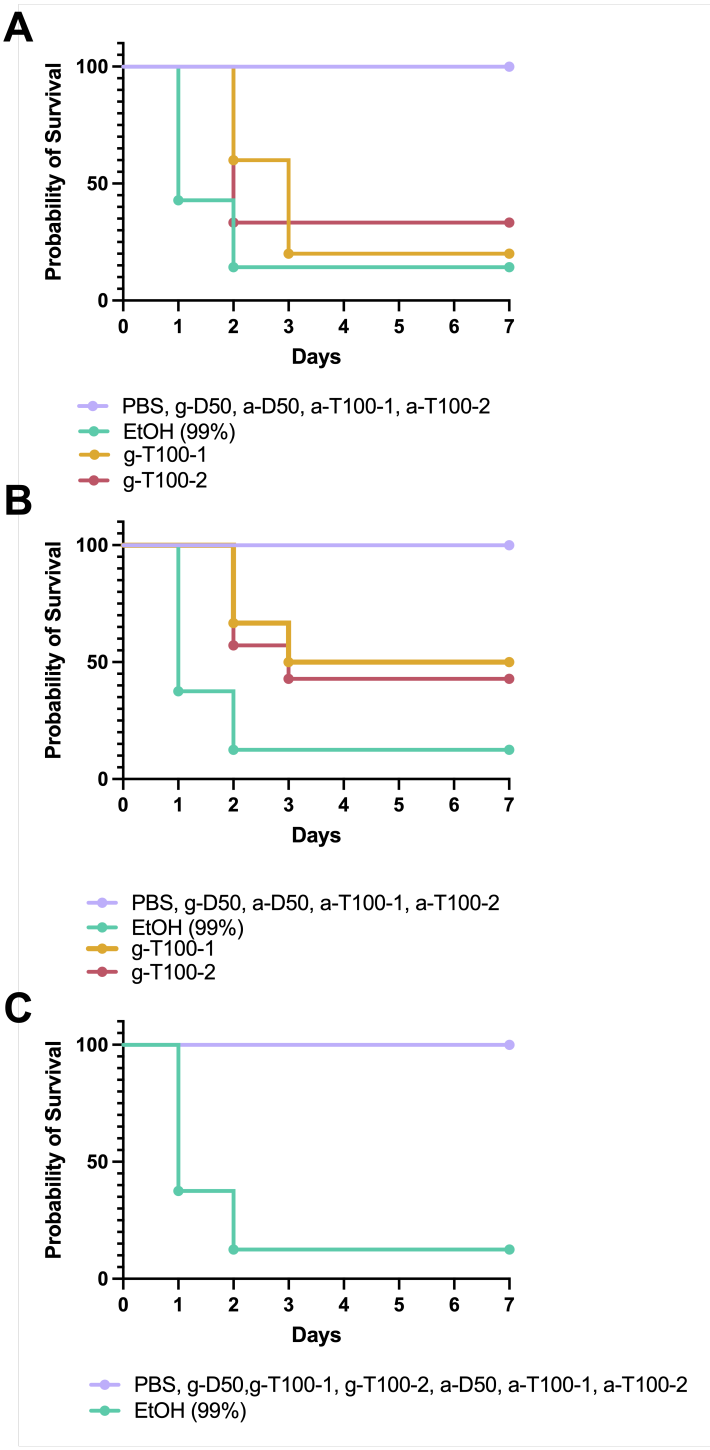

Figure S20. Survival plot of *Galleria mellonella* injected with the copolymers (**A**) at 1024 µg mL^{-1}, (**B**) 512 µg mL^{-1} and (**C**) 256 µg mL^{-1}. 8 larvae were used in each conditions and they were monitor for 7 days.

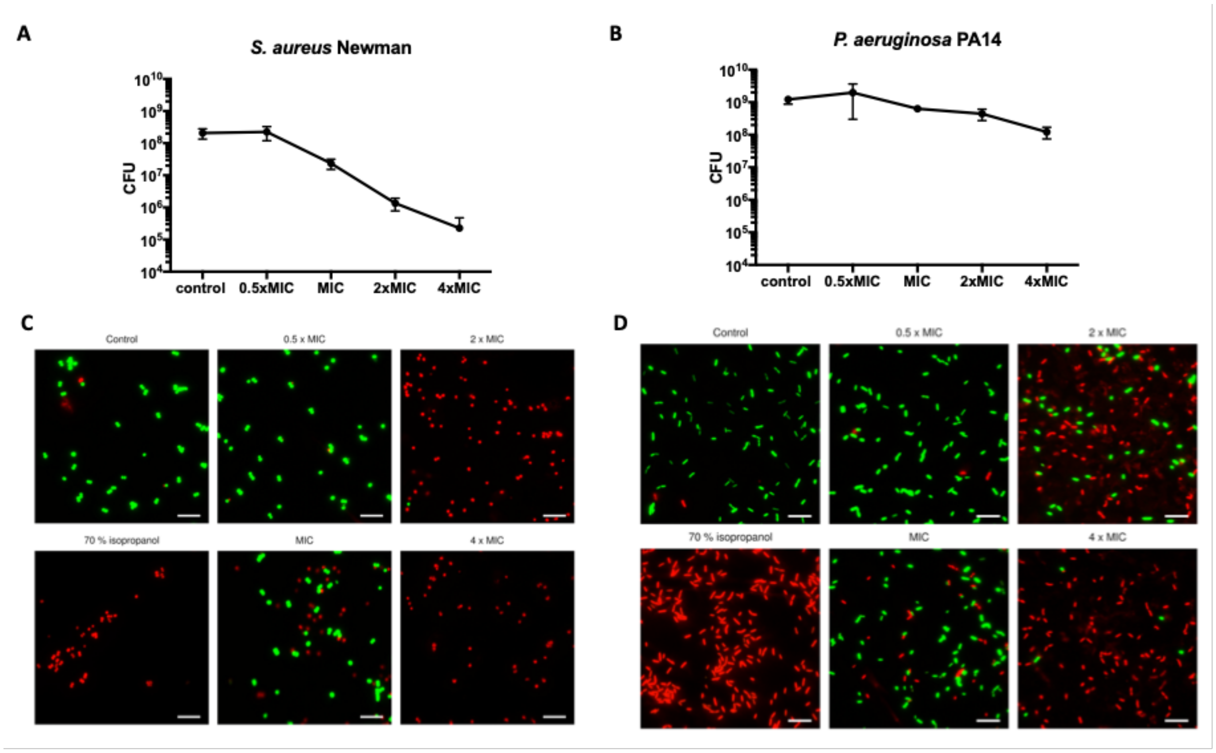

Figure S21. (A) Colony count forming units from $OD_{600} = 0.5$ of *S. aureus* Newman in caMHB after 1h control, 0.5 x MIC, MIC, 2 x MIC and 4 x MIC of g-D50. (B) Colony count forming units from $OD_{600} = 0.5$ of *P. aeruginosa* PA14 in caMHB after 1 h control, 0.5 x MIC, MIC, 2 x MIC and 4 x MIC of a-T100-1. Shown are the averages of three biological replicates ± standard error. (C) Fluorescence microscopy images of live/dead stained *S. aureus* Newman cells treated with 0.5 x MIC, MIC, 2 x MIC and 4 x MIC of g-D50. (D) Fluorescence microscopy images of live/dead stained *P. aeruginosa* PA14 cells treated with 0.5 x MIC, MIC, 2 x MIC and 4 x MIC of a-T100-1. Living cells are stained in green (SYTO9 dye), while dead cells are stained in red (propidium iodide). Control cultures were not treated with any compound. Cultures treated with 70 % isopropanol were included to demonstrate 100 % compromised cells. Scale bar: 10 µm.

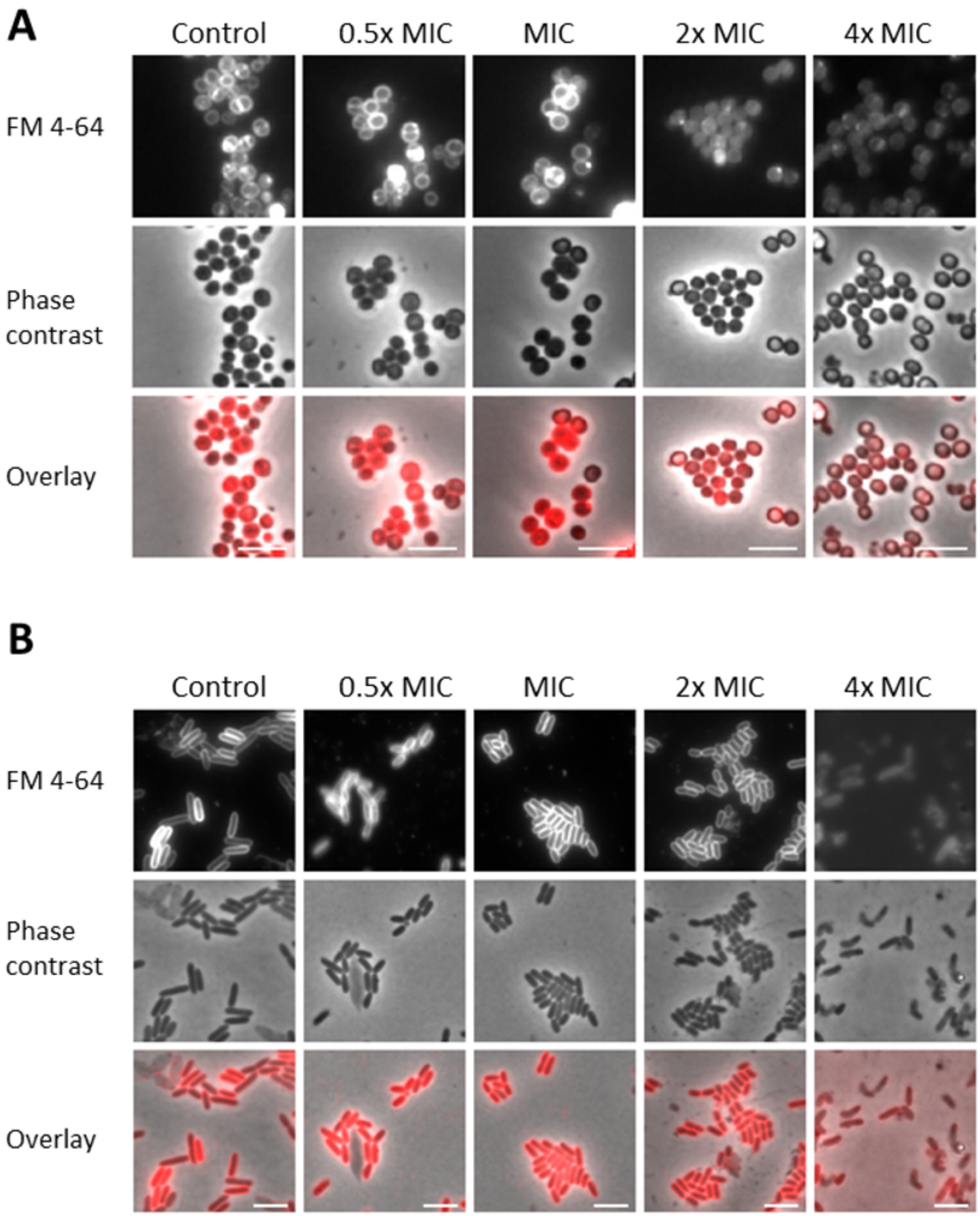

Figure S22: The effect of treatment of (A) *S. aureus* Newman with g-D50 and (B) *P. aeruginosa* PA14 with a-T100-1 on membrane staining of the lipophilic dye FM4-64 FX. The compounds were added at the indicated final concentration for 30 min prior to staining with the dye, and fixation. Scale bar: 4 μm.

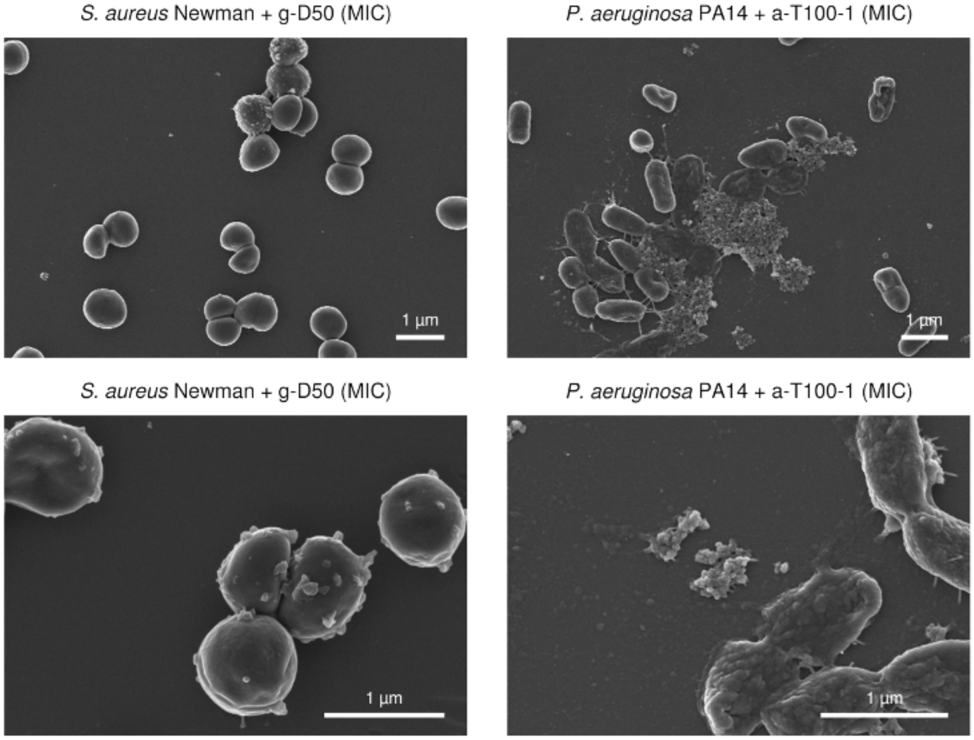

Figure S23. Scanning electron micrographs of *S. aureus* Newman exposed to g-D50 (MIC concentration) and *P. aeruginosa* PA14 exposed to a-T100-1 (MIC concentration).

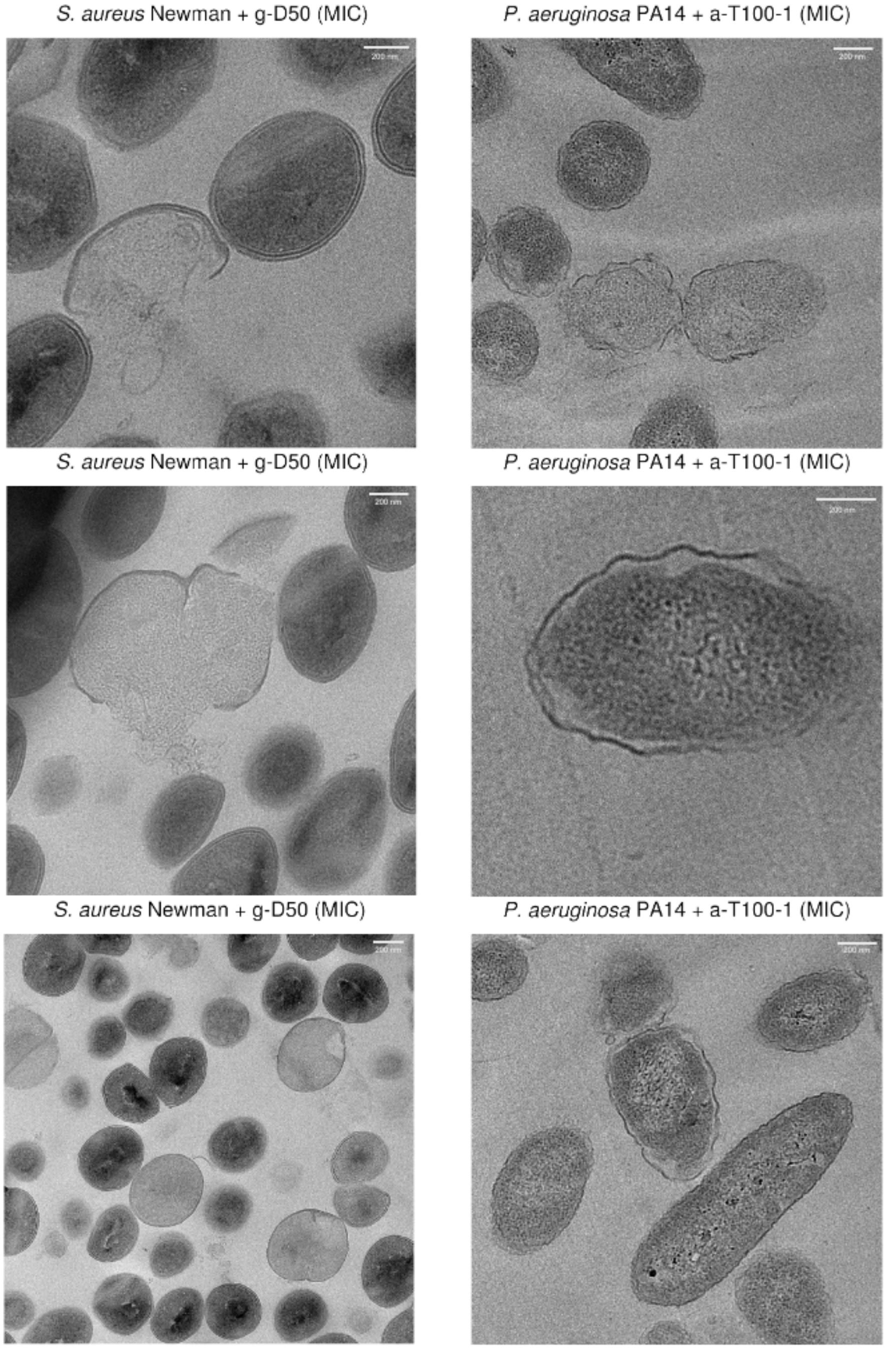

Figure S24. Transmission electron micrographs of *S*. *aureus* Newman treated with g-D50 (MIC concentration) and *P*. *aeruginosa* PA14 treated with a-T100-1 (MIC concentration).

REFERENCES

(1) Martin, L.; Peltier, R.; Kuroki, A.; Town, J. S.; Perrier, S. Investigating Cell Uptake of Guanidinium-Rich RAFT Polymers: Impact of Comonomer and Monomer Distribution. *Biomacromolecules* **2018**, *19* (8), 3190-3200. DOI: 10.1021/acs.biomac.8b00146.
(2) Hobson, L. J.; Feast, W. J. Poly(amidoamine) hyperbranched systems: Synthesis, structure and characterization. *Polymer* **1999**, *40* (5), 1279-1297. DOI: 10.1016/S0032-3861(98)00268-7.
(3) Arnold, O.; Bilheux, J. C.; Borreguero, J. M.; Buts, A.; Campbell, S. I.; Chapon, L.; Doucet, M.; Draper, N.; Ferraz Leal, R.; Gigg, M. A.; et al. Mantid—Data analysis and visualization package for neutron scattering and μ SR experiments. *Nuclear Instruments and Methods in Physics Research Section A: Accelerators, Spectrometers, Detectors and Associated Equipment* **2014**, *764*, 156-166. DOI: 10.1016/j.nima.2014.07.029.
(4) King, S. M. Small-angle neutron scattering. In *Modern Techniques for Polymer Characterization*, John Wiley & Sons, 1999; pp 171-232.
